# Enhancing the visual environment of urban coastal roads through deep learning analysis of street-view images: A perspective of aesthetic and distinctiveness

**DOI:** 10.1371/journal.pone.0317585

**Published:** 2025-01-14

**Authors:** Yu Zhang, Xing Xiong, Shanrui Yang, Qinghai Zhang, Minghong Chi, Xiaoyu Wen, Xinyu Zhang, Junwei Wang

**Affiliations:** 1 Department of Landscape Architecture, Nanjing Agricultural University, Nanjing, China; 2 Academy of Fine Arts, Jiangsu Second Normal University, Nanjing, China; CNESTEN: Centre National de l’Energie des Sciences et des Techniques Nucleaires, MOROCCO

## Abstract

Urban waterfront areas, which are essential natural resources and highly perceived public areas in cities, play a crucial role in enhancing urban environment. This study integrates deep learning with human perception data sourced from street view images to study the relationship between visual landscape features and human perception of urban waterfront areas, employing linear regression and random forest models to predict human perception along urban coastal roads. Based on aesthetic and distinctiveness perception, urban coastal roads in Xiamen were classified into four types with different emphasis and priorities for improvement. The results showed that: 1) the degree of coastal openness had the greatest influence on human perception while the coastal landscape with a high green visual index decreases the distinctiveness perception; 2) the random forest model can effectively predict human perception on urban coastal roads with an accuracy rate of 87% and 77%; 3) The proportion of low perception road sections with potential for improvement is 60.6%, among which the proportion of low aesthetic perception and low distinctiveness perception road sections is 10.5%. These findings offer crucial evidence regarding human perception of urban coastal roads, and can provide targeted recommendations for enhancing the visual environment of urban coastal road landscapes.

## 1 Introduction

Urban waterfront areas integrate nature, culture, and society, and are renowned for their aesthetic appeal and distinctiveness [1]. They serve as significant exhibition spaces that accentuate a city’s dynamism and unique characteristics [2]. Desirable urban waterfront areas can enhance the quality of the urban environment, cultivate cultural experiences, and promote tourism development [3]. Urban waterfront spaces consist of both natural and artificial landscape elements [4]. The quality of these spaces influences the style and features of the urban landscape, public behavior [5,6], and physical and mental health [7]. Several studies have discussed the impact of urban waterfront spaces on public aesthetics and emotions.

Urban coastal roads, as fundamental linear elements of urban waterfront areas, are some of the most attractive and charming characteristic spaces in a city, and they possess significant recognizability. Urban style and features can express the external image of a city. High-quality environments along coastal roads can promote tourism and economic development. Blue-green spaces play a crucial role in promoting health themes. Nowadays, high-quality waterfront landscapes significantly enhance the aesthetic value and allure of urban landscapes, with their planning and construction increasingly prioritized for human perceptual experiences [8–11]. Therefore, how to highlight the features of waterfront streetscapes, harmonize the relationships among landscape elements, and meet the public’s aesthetic needs has become an important focus of research on human living environments.

Perception is the process of attaining awareness or understanding of sensory information. With the development of environmental psychology and cognitive theories, landscapes are regarded as integrations of objective spaces and human cognitive perceptions [12]. The field of landscape perception has a long history of research aimed at understanding how individuals perceive and interact with various landscapes [13,14]. It is widely acknowledged that visual perception predominantly influences landscape experiences through individual perceptions of various landscape elements, each equally important in shaping the overall experience [15]. Public perception of road quality is related to landscape elements. This depends on the physical components of the streetscape [16], such as buildings, fences, greenery, road width, pedestrian spaces, motorization, and the sky [17–19]. Physical features are an interesting and important part of or feature of the visible street elements. These physical features can quantify the road environment based on visual information, such as building-to-land ratio, greenness, fence area, height-to-width ratio, street scale, and cleanliness [20]. The five physical features of imageability, enclosure, human scale, transparency, and complexity are considered by researchers as potential indicators of urban design quality [21–25].

To bridge the connection between human perception and the objective environment in landscapes, scholars have employed a variety of methods, ranging from surveys and field observations to advancements in neuroscience technology [26]. These approaches aid researchers in exploring the complex interactions between human cognition, emotional responses, and their combined impact on individual aesthetic evaluations, ultimately elucidating the intricate dynamics underlying landscape perception [2,27,28]. Street view images (SVI) have emerged as a primary data source because of their high accessibility, high resolution, and comprehensive coverage [29]. These geotagged images offer unprecedented opportunities for large-scale and in-depth studies of urban street perception [30]. Some researchers have attempted to integrate deep learning techniques with big data, utilizing computer vision technology to quantitatively analyze massive street view images, thereby reflecting individuals’ perceptions of various locations [31,32]. Compared with traditional machine learning methods, deep learning, which incorporates technologies like Convolutional Neural Networks (CNNs), Recurrent Neural Networks (RNNs), and Generative Adversarial Networks (GANs), minimizes the necessity for human intervention, thus increasing work efficiency [33,34]. Various image segmentation techniques like Fully Convolutional Network (FCN), Pyramid Scene Parsing Network (PSPNet), and Segmentation Network (SegNet) leverage CNNs to process visual information in images, enabling the accurate identification of multiple features like roads, vegetation, buildings, vehicles, and water bodies [35,36]. SVIs effectively represent and measure visual landscape features, including in urban waterfront areas where their relationship with visual perception has been explored using image semantic segmentation [37], and the characteristics of urban waterfront areas have been evaluated in conjunction with virtual reality technology [38]. Recently, emerging machine learning techniques such as Random Forest (RF), Support Vector Machine (SVM), and Naive Bayes (NB) have been applied to train models using extensive data to extract features from street view images automatically. These techniques can be utilized to predict a broad spectrum of environmental perceptions [39,40]. Image semantic segmentation based on deep learning has been widely applied, utilizing information recognition, feature extraction, and classification to perform detailed quantification and analysis of scene semantics. This provides landscape architects with valuable visual insights and decision support.

Perceptual evaluation, being a subjective evaluation, typically employs public preference surveys to enhance the effectiveness of the evaluation [41–43]. An essential method for evaluating urban aesthetic quality is the visual inspection of the physical indicators of urban landscapes, either independently or in combination with other landscape elements [44,45]. Scenic Beauty Estimation (SBE) [46], the Law of Comparative Judgment (LCJ), and Semantic Differentiation (SD) are examples of subjective psychophysical methods. The SBE method has been frequently adopted to quantify public preferences for riverfront aesthetics, integrating subjective evaluations with objective scenery features, enabling the analysis of influential factors and providing a framework to understand the aesthetics and value of these environments. The aesthetics of urban open spaces serve as potential contributors to the development of the waterfront landscape [47]. Recent studies have revealed the aesthetic perception of urban waterfront areas as intricately tied to various elements such as buildings, fences, trees, roads, motorization, naturality, and landscape diversity [48,49].

Urban distinctiveness, as sensory features perceived by humans, can transcend intuitive to rational comprehension, showcasing a city’s unique style and distinctiveness [50]. Building form and color play a significant role in shaping human perception of urban characteristics, with urban waterfront areas’ distinctiveness largely defined by their streets, landmarks, and nodes [51]. Methods of landscape aesthetic evaluation can also be extended to the assessment of landscape distinctiveness, allowing for the evaluation of the visual quality of urban environments.

Based on the above studies, this study utilizes SVIs and employs deep learning techniques, and questionnaires to assess the multidimensional perception along the urban coastal roads in Xiamen. Our primary objective was to address the following three questions:(1) What landscape features significantly affect the aesthetic and distinctiveness perception of urban coastal roads? (2) How to establish an effective model for representing and predicting the perception of aesthetic perception and distinctiveness perception of urban coastal roads? (3) How to enhancing the visual environment of urban coastal roads based on aesthetic and distinctiveness perception?

## 2 Materials and methods

### 2.1 Study area

**Fig 1:**
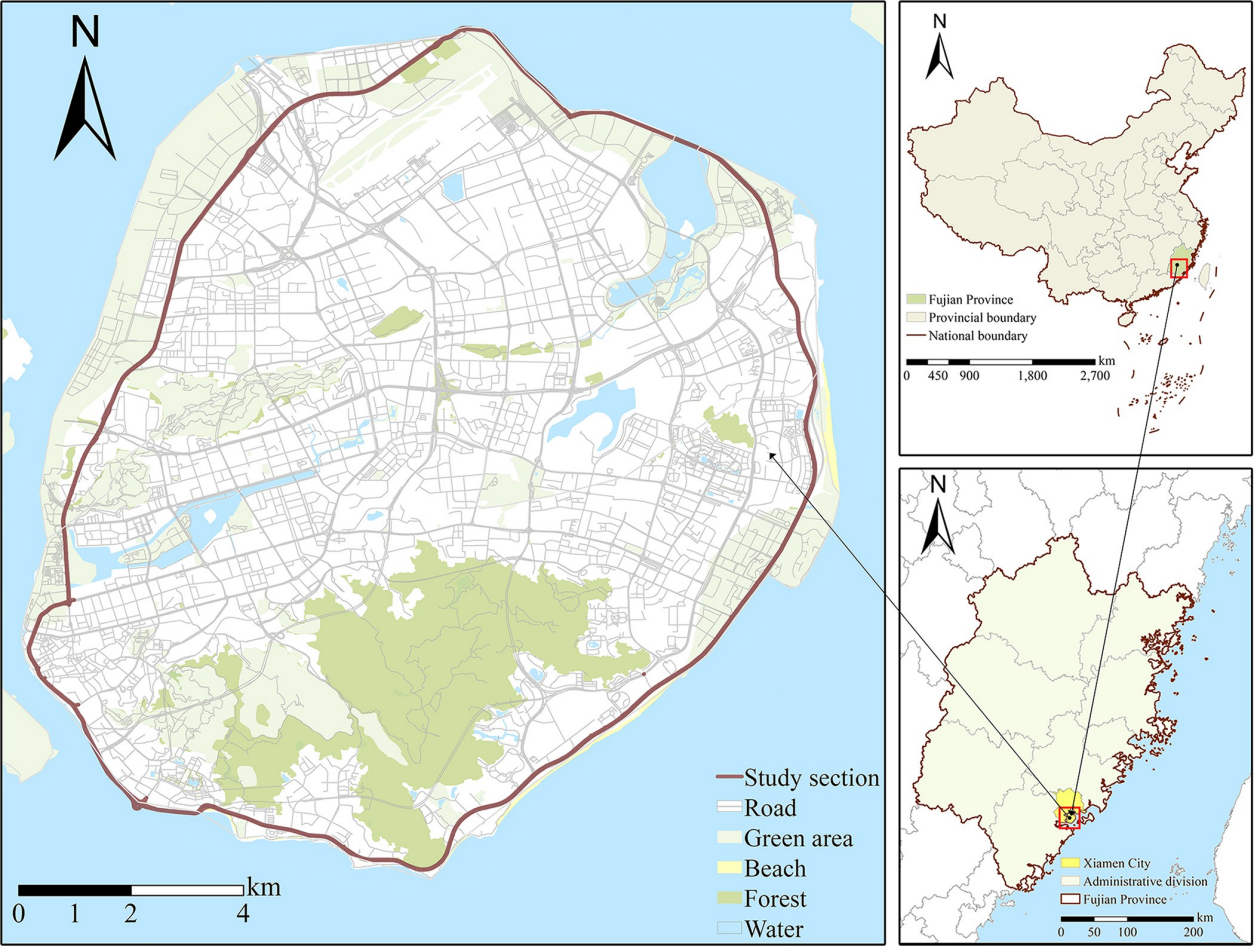
Map of the study area.

Xiamen located in Fujian Province on the southeast coast of China, featuring natural landscape of mountains and oceans. In the urban planning of Xiamen, the city is strategically positioned as a maritime garden city due to its unique geographical advantages and rich coastal natural and cultural landscapes. The most attractive and charming distinctive of Xiamen is the coastal road space, which obviously identifiable. The study area is the Island Ring Boulevard in Xiamen, an urban coastal scenic road on Xiamen Island. The Island Ring Boulevard seamlessly connects the natural and cultural landscapes along the seashore, effectively showcasing the distinctive subtropical scenery of Xiamen. The site covers a total distance of 43 km, passing through the Wuyuan Bridge, Huandao East Road, Huandao South Road, Yanwu Bridge, Lujiang Road, Hubin West Road, Dongdu Road, Chang’an Road, Airport North Ring Road, Airport North Road, and Huandao North Road (Fig 1).

### 2.2 Research framework

**Fig 2:**
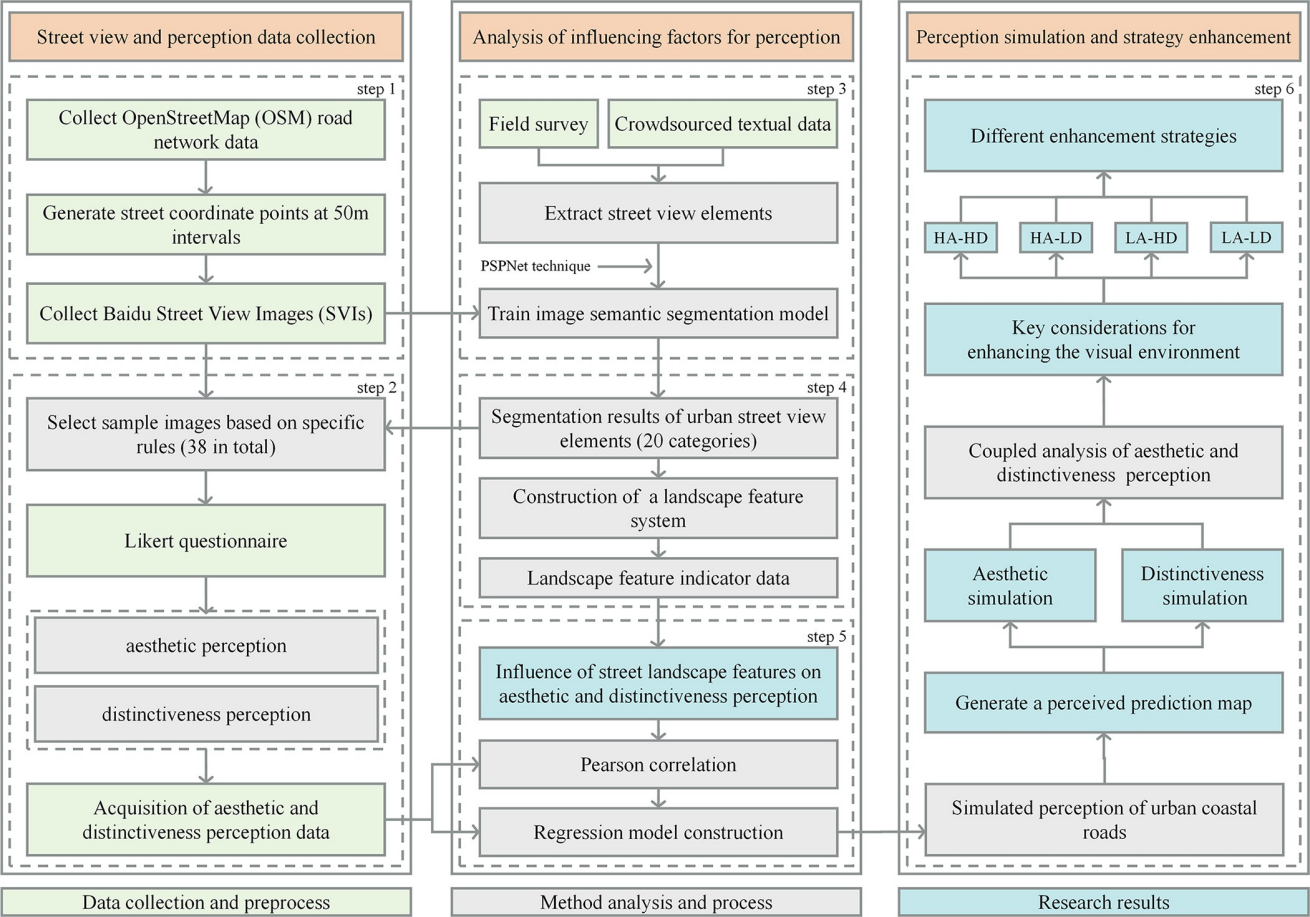
Research framework.

The study consisted of three primary stages, as shown in Fig 2.

In the first stage, we collected urban street view images and data on aesthetic and distinctiveness perception scores. Specifically, we gathered Baidu SVIs of the street network in the study area. We selected sample images from these Baidu SVIs based on specific rules to perform aesthetic and distinctiveness perception. We obtained perceptual evaluation scores through questionnaires.

In the second stage, we evaluated the aesthetics and distinctiveness of urban coastal roads. We first extracted street view elements of urban coastal roads through field research and crowdsourced data. Using the PSPNet algorithm, we constructed a neural network model for image semantic segmentation, enabling full-feature segmentation of Baidu SVIs data. Additionally, we developed a landscape feature index system for aesthetic and distinctiveness perception. Based on the data obtained from image segmentation, we calculated the landscape feature data for all images within the study area. We then used statistical analysis to determine the impact of different landscape feature levels on aesthetic and distinctiveness perception. Furthermore, we attempted to construct regression models for aesthetic and distinctiveness perception.

In the third stage, we proposed strategies to enhance the landscape of urban coastal roads. We used regression models to predict the levels of aesthetic and distinctiveness perceptions along urban coastal roads. By integrating aesthetic and distinctiveness perceptions, we classified coastal roads into different categories. Finally, for each road category, we proposed various strategies aimed at enhancing the visual landscape of coastal urban roads.

### 2.3 Baidu street view images collection

**Fig 3:**
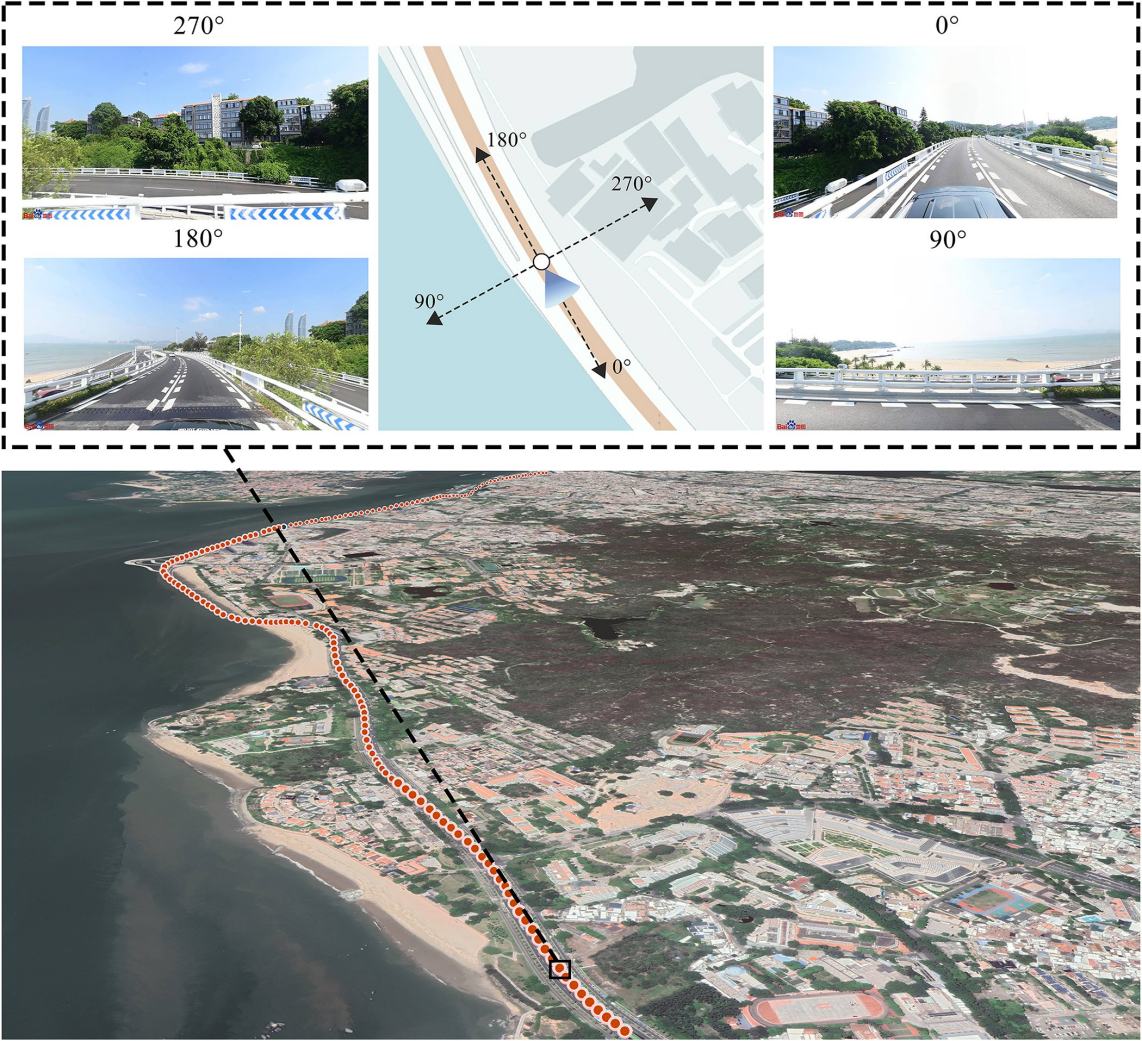
Schematic of the Baidu SVI collection.

Online SVIs from Google, Baidu, and Tencent provide more comprehensive, reliable, and specific data support for research on the visual quality of larger cities. Baidu Map (https://map.baidu.com/), one of the largest online maps that allows users to obtain static SVIs from different perspectives, was selected as the main data source for obtaining SVIs [52]. The road network data and map data for Island Ring Boulevard were sourced from Open Street Map (OSM) website (OSM: https://www.openstreetmap.org; China road network: https://download.geofabrik.de/asia.html). Based on the visual distance from the perspective of road travel and previous research, we selected a standard interval of 50 m and generated 1540 sampling points using ArcGIS software. We then obtained SVIs from the Baidu Map open platform using the application programming interface. We set the pitch angle of each sampling point to 0° (horizontal) and captured SVIs in four directions (front, right, back, and left) at 0°, 90°, 180°, and 270° in the horizontal direction. Finally, we use the images of these four perspectives to create individual sampling point data (Fig 3). The update time of street view data in the study area is from April 2020 to March 2021. Because Xiamen is located in a subtropical climate region, where the streets are dominated by evergreen tree species, the seasonal variations in the streetscape can be ignored [53]. In total, 1340 points were captured, yielding 5360 valid SVIs, each with a size of 1024 × 512 pixels.

### 2.4 Scoring aesthetic and distinctiveness perception

**Fig 4:**
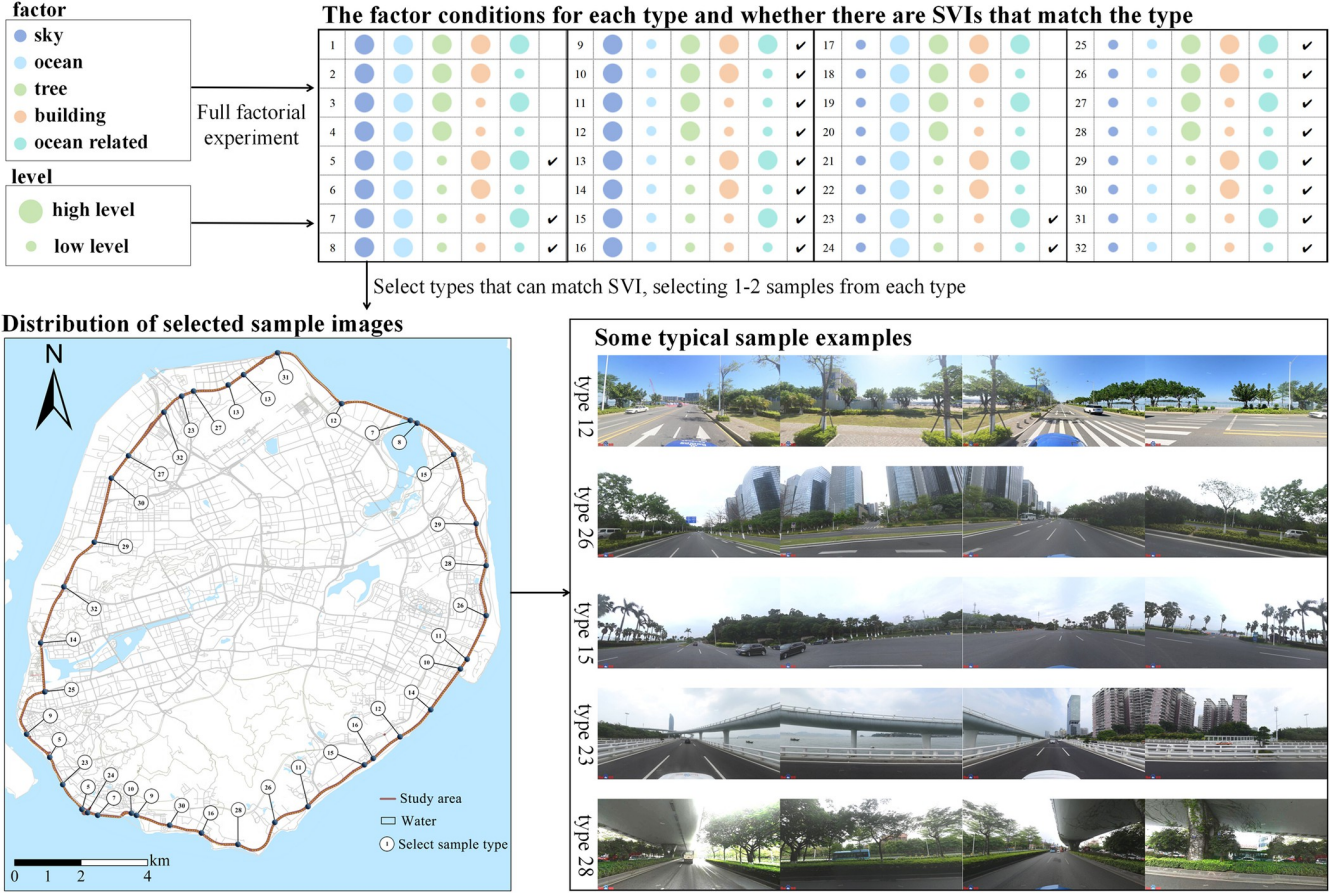
Questionnaire sample selection process and sample image distribution map.

We employed Baidu SVIs as a reliable measure of individuals’ restorative perception, as previous studies have shown the suitability of photo graphs as accurate substitutes for real environments [54]. In this study, we employed a factorial design methodology to systematize the selection of sample images, ensuring comprehensiveness and representativeness [55]. Initially, we identified the five factors with the highest occupancy rates in the images, specifically sky, plants, buildings, ocean, and urban feature elements (including palm plants, islands, beaches, landmarks, and bridges), while excluding roads. These elements were then classified into two levels, high and low, based on their proportion in the images, using the natural breakpoint method. Based on these visual elements and their proportion levels, we developed a sample selection framework that incorporates all possible combinations. Through this factorial design, we generated 32 distinct sample types [55]. Based on the results of the semantic segmentation of images, we matched the SVI set with these types, excluding some types that lacked data, such as Type 1, ultimately obtaining 21 valid types. Next, we selected 1–2 typical images from each type, ensuring that the total selected images were relatively evenly distributed within the scope of this study. This ensures that the samples can adequately represent the landscape feature of each road section (Fig 4). Finally, 38 typical sample points were selected as questionnaire sample data to further explore the relationship between perception and landscape features. The design ensures that the selected image samples extensively represent the visual diversity of the study area, thereby enhancing the generalizability and accuracy of the research findings.

To gauge the perception of roads, we developed a 7-point Likert scale questionnaire on the Wenjuanxing platform (https://www.wjx.cn/) [56]. The questionnaire includes two dimensions: aesthetic and distinctiveness perception. The survey was carried out from July 1, 2023 to September 30, 2023. The “Ethical Committee” of Nanjing Agricultural University approved the study. A verbal consent was obtained from the participants of the study. During the experimental phase, a total of 1,649 participants were asked to rate two dimensions for 38 selected sample images online. Each participant rated the images, leading to a total of 1,638 valid questionnaires collected, resulting in 62,244 score items. To ensure fairness and consistency in scoring, the image order in each questionnaire is randomized. Participants were required to rate each question from 1 to 7, indicating the extent to which their actual perceptions matched the images they viewed.

### 2.5 Deep learning-based semantic segmentation of SVIs

**Fig 5:**
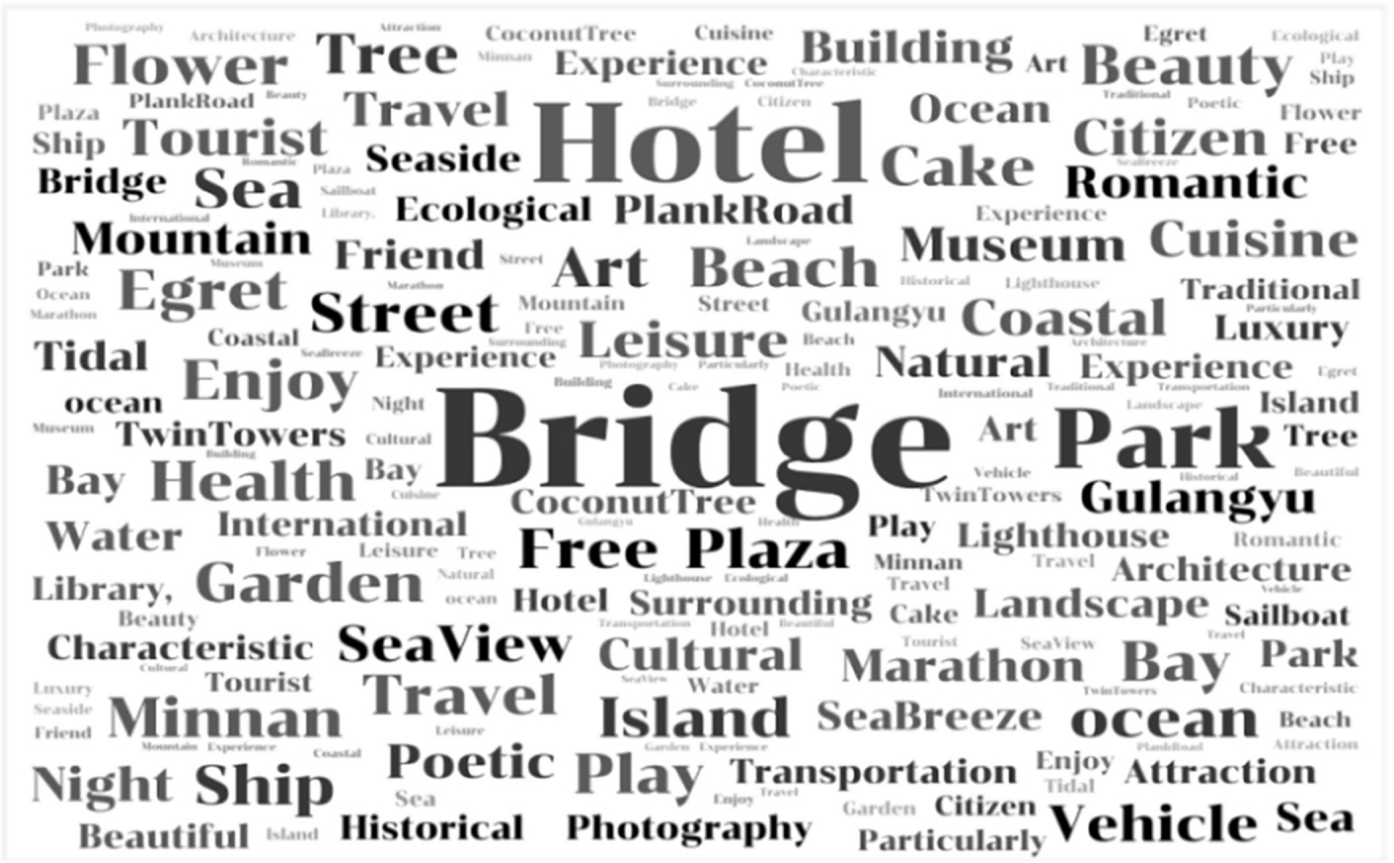
Word frequency analysis of crowdsourced textual data.

Before image segmentation, we identified key elements using online review texts with the aim of comprehensively capturing the essence of urban coastal road landscapes. We used Octopus software to collect Island Ring Boulevard-related online review texts from Ctrip for feature elements extraction of Xiamen coastal area. The collected network text of Xiamen Island Road was imported into ROST-CM6 software for segmentation and quantification of vocabularies and sentences [52]. Based on high-frequency words and word frequency, a word cloud map (Fig 5) was used to visually present the public’s overall perception of Xiamen Island Ring Boulevard. The larger the font on the map, the higher the attention of tourists.

Combining online review texts with research on street view features, we extracted 20 types of landscape elements categorized into two main groups: road skeleton (sky, plants, grass, mountains, roads, sidewalks, walls, buildings, railings, cars, people, bicycles, and facilities) and regional characteristics (ocean, ships, islands, palm plants, beaches, landmarks, bridges).

We used a deep learning-based image semantic segmentation model to extract landscape elements from the SVIs of urban coastal roads, thereby laying the foundation for quantitative measurement research on coastal road environments. After comparing the performance of various models, we selected PSPNet due to its superior accuracy among segmentation models such as FCN8s, SegNet, and PSPNet [57–59]. PSPNet fully uses global feature prior knowledge to analyze different scenes and achieve semantic segmentation of scene target [60]. For scene parsing and semantic segmentation tasks, PSPNet extracts appropriate global features, uses pyramid pooling modules to fuse local and global information, and proposes an optimization strategy with moderate supervised loss, which has good performance.

**Fig 6:**
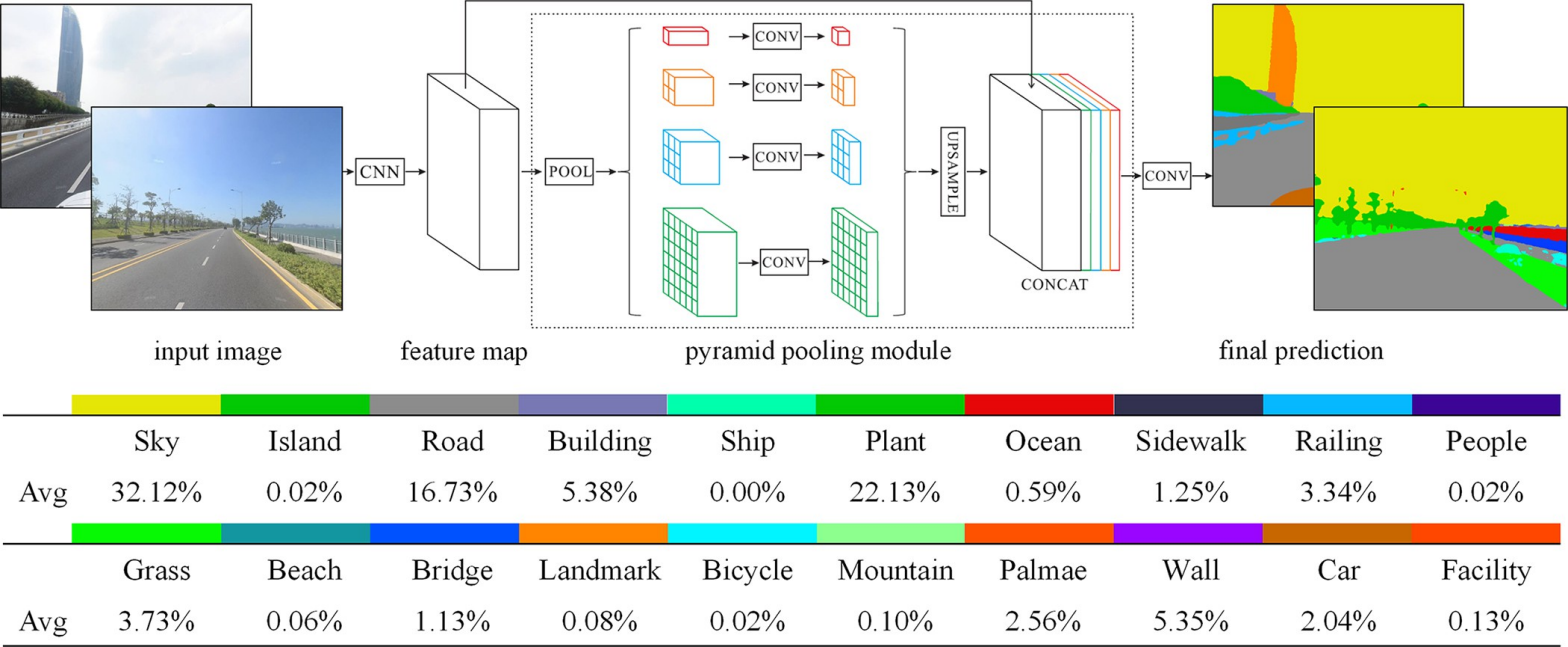
Comparison of an image before and after semantic segmentation.

Due to the current dataset’s inability to cover all the elements we needed, especially the absence of landmarks and islands, we collected 1,638 Baidu SVIs of Xiamen Island as a supplementary dataset. We used transfer learning to adjust the ADE20K dataset and trained a PSPNet semantic segmentation model for coastal road landscapes (Fig 6). Statistics were compiled on the average proportion of each element.

During the training process, the semantic segmentation model achieved overall pixel accuracy of 0.91, and the mean intersection-to-union ratio was 0.79, indicating that the semantic model can accurately process batches of coastal road images. The accuracy of each element is above 0.87, with the highest reaching 0.96, and the intersection-to-union ratio is higher than 0.64. With this level of accuracy, the image semantic segmentation model demonstrates high recognition rates for each label category, making it applicable to the visual landscape of urban coastal roads (Table 1).

**Table 1:** Segmentation accuracy of each element in the semantic model.

No.	Category	PA	MIoU	No.	Category	PA	MIoU
1	sky	0.942	0.961	11	bridge	0.922	0.796
2	plant	0.914	0.874	12	wall	0.931	0.639
3	island	0.892	0.638	13	building	0.933	0.899
4	grass	0.936	0.764	14	railing	0.878	0.639
5	mountain	0.911	0.972	15	landmark	0.906	0.659
6	ocean	0.956	0.912	16	car	0.901	0.634
7	beach	0.936	0.865	17	ship	0.928	0.596
8	palm plant	0.914	0.826	18	people	0.936	0.699
9	road	0.963	0.957	19	bicycle	0.916	0.706
10	sidewalk	0.912	0.639	20	facility	0.915	0.599

PA (Piexl Accuracy) = 0.91; MIoU (Mean Intersection over Union) = 0.79.

### 2.6 Construction of a landscape feature system

Landscape vision is a relationship between seeing and being seen that is constituted under certain conditions by the human-centered viewer as the subject and various landscapes as objects [61]. On the basis of previous research and the feature of the urban coastal landscape, We constructed feature indicators of the coastal roads (Tables 2 and 3).

**Table 2:** Calculation of visual feature indicators for coastal road landscape.

Indicators	Formula	Indicators	Formula
green visual index (GVI)	$GVI=P_{pl}+P_{gr}+P_{pa}$	color diversity (CD)	$CD=-\sum_{i=1}^{n}CP_{i}\mathrm{lg}P_{i}$
building visibility (BV)	$BV=P_{bu}+P_{la}$	blue visual index (BVI)	$BVI=P_{oc}$
landscape complexity (LC)	$LC=-\sum_{i=1}^{n}P_{i}\mathrm{lg}P_{i}$	marine-related element index (MREI)	$MREI=P_{sh}+P_{br}+P_{be}+P_{is}$
interference factor index (IFI)	$IFI=P_{fa}$	characteristic plant visibility (CPV)	$CPV=P_{pa}$
coastal openness (CO)	$CO=P_{oc}+P_{sk}$	landmark visibility (LV)	$LV=P_{la}$

Notes: Ppl denotes the pixel of plants (including trees and shrubs) in the image, Pgr denotes the pixel of grass in the image, Ppa denotes the pixel of palm plants in the image, Pbu denotes the pixel of buildings in the image, Pla denotes the pixel of landmarks in the image, Pfa denotes the pixel of facilities in the image, Poc denotes the pixel of ocean in the image, Psk denotes the pixel of sky in the image, Psh denotes the pixel of ships in the image, Pbr denotes the pixel of bridges in the image, Pbe denotes the pixel of beaches in the image, Pis denotes the pixel of islands in the image, and C represents the color factor.

**Table 3:** Selection criteria for visual characteristic indicators of coastal road landscape.

Indicators	Indicator selection criteria
GVI	Green is the most significant landscape element in road landscapes, affecting the ecological and natural levels of coastal spaces. The green vision rate is an indicator of the human perception of the surrounding green environment, which has a crucial impact on visual attraction.
BV	The density of coastal buildings is a manifestation of coastal space development and construction, and the visibility of buildings has an impact on the quality of coastal visual perception.
LC	Using visual entropy to represent the landscape complexity index, the more types of elements in the environment, the higher the visual entropy.
IFI	Warning signs, trash cans, streetlights, and other artificial facilities are dynamic landscape and service elements in coastal vision. Their existence can influence the overall judgment of the human visual perception of the landscape at a certain point, affecting the sense of order in the landscape.
CO	Openness is a fundamental attribute of urban waterfront space, and the visibility of the sky determines perceived brightness, which has an impact on visual perception and pleasure. The sky and the water surface are the main manifestations of natural openness.
CD	Characterizing the richness of various colors in the road, the more types of elements in the road landscape, the higher the level of color stratification and richness
BVI	The water body is the main element in coastal roads, and as a visual design element, it has a critical visual influence. The public usually prefers spaces with water.
MREI	The landscape elements with significant features or distinguishability in coastal areas are easy to pay attention to, remember, and extract, and they also help establish a visual connection between the city and the coastal area for the public.
CPV	Plant communities with distinct regional feature are conducive to strengthening the human perception of specific areas.
LV	Urban landmarks, which integrate significant natural and cultural elements of the city, are places that reflect and represent the overall feature of the city. They serve as a microcosm of the city, affecting the public’s recognition of the city.

### 2.7 Perception influencing factors and model construction

To explore the correlation between various landscape features and perceptions of aesthetics and distinctiveness, we employed Pearson correlation analysis. Pearson correlation is the most commonly used method to measure linear correlations. When utilizing Likert scales with four or more categories, Pearson correlation remains very stable for results that violate assumptions, making it suitable for verifying correlations [62].

For the predictive models, we selected the stepwise Ordinary Least Squares (OLS) regression model and the random forest model. The results of the two models can mutually verify each other, with the model demonstrating superior fit selected to predict perceptions across the entire study area.

Using the stepwise OLS regression model, we effectively established a linear relationship model to predict and explain the public’s visual scoring of landscapes. Stepwise OLS is a variable selection technique that iteratively adds or removes predictive variables based on statistical criteria such as the Akaike Information Criterion (AIC) or Bayesian Information Criterion (BIC), identifying variables that most influence the model’s predictions of public visual scoring. Starting with a basic model that includes a minimal number of predictive variables, usually chosen based on prior knowledge or importance estimation, this method effectively reduces model complexity while avoiding overfitting by adding or removing variables and assessing improvements at each step. Furthermore, we addressed the issue of multicollinearity in the model by calculating the Variance Inflation Factor (VIF) to diagnose the strength of linear relationships among independent variables. Ultimately, the parameters of the stepwise OLS model were estimated using the standard least squares method, ensuring unbiased estimations and minimal variance.

Employing a decision tree based on the Classification and Regression Tree (CART), the RF model is an ensemble learning method based on bagging [63]. It generates multiple models for independent learning and prediction, then aggregates the results of each model and adopts the voting outcomes of multiple decision trees as the final single prediction [64]. It exhibits good tolerance to interference and is used in regression problems [63]. We incorporated all physical features of the landscape as independent variables and the public’s visual scoring of the landscape as the dependent variable. To ensure the accuracy and generalization ability of the model, we utilized the GridSearchCV module to find the most suitable parameter set. GridSearchCV systematically searches over a specified parameter grid, evaluating the model performance using cross-validation to identify the optimal combination of hyperparameters. In this study, we defined a range of hyperparameters for the Random Forest model, such as the number of trees, the maximum depth of the trees, and the minimum number of samples required to split a node. By evaluating all possible combinations of these parameters, we identified the best set that maximized the model’s performance. Additionally, we employed the N-fold method to further limit the minimum number of sample divisions and leaf node samples. We also used the maximum feature value and other parameters to ensure the model’s generalization capability. We divided 62,244 score items into two groups: the independent variable set χ, representing the proportions of 10 landscape features for each sample image, and the dependent variable set γ, representing the corresponding restorative perception scores. We split χ and γ into training and testing sets, with a training ratio of 70% and a testing ratio of 30%.

### 2.8 Perception simulation and coupling analysis

We mapped the perceptual scores of the regression model to seven levels ranging from 1 to 7. These scores were then mapped onto the road network. We used the Natural Breaks method to classify predictions of aesthetics and distinctiveness. Aesthetic predictions were categorized into high aesthetics (HA) and low aesthetics (LA), while distinctiveness predictions were divided into high distinctiveness (HD) and low distinctiveness (LD). Based on these classifications, coastal roads were assessed, and a four-quadrant matrix was constructed using two dimensions: aesthetic perception of coastal roads (HA and LA) and distinctiveness perception of coastal roads (HD and LD). We used this matrix to identify features that need improvement on different coastal roads. Subsequently, we conducted targeted discussions on the perceptual characteristics and enhancement potential of each category of coastal roads.

## 3 Result

### 3.1 Impact of landscape features on aesthetic and distinctiveness perception

According to the results of the questionnaire, the average score of the aesthetic perception of the sample images is 4.70/7.00, and the average score of the distinctiveness perception is 4.69/7.00.

**Fig 7:**
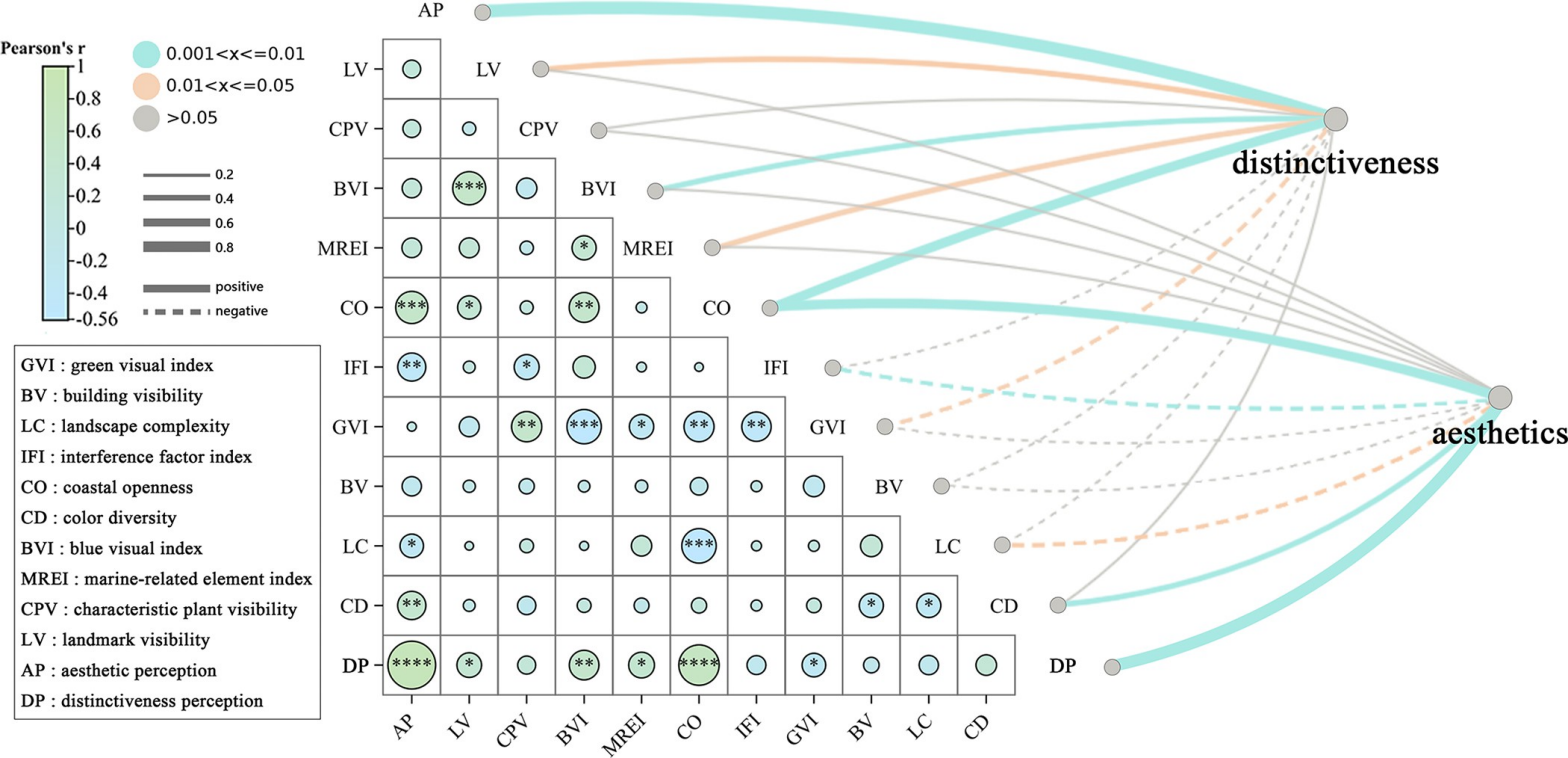
Correlation between landscape features and human perception.

The correlation between landscape features and human perception of different categories is illustrated in Fig 7. In the correlation heatmap, the tow points on the right represent aesthetics and distinctiveness perception. The width and color of the connecting parts represent the statistical values of the correlation coefficient r and the significance p value and the positive or negative correlation relationship.

In this study, correlation analysis revealed significant associations between Aesthetic Perception (AP) and Distinctiveness Perception (DP) and various environmental features. Based on the correlation analysis results, there is a very high correlation between aesthetic perception and distinctiveness perception (r = 0.852). This suggests that they may share some common environmental influences. However, different landscape features play distinct roles in aesthetic and distinctiveness perception evaluations, and the extent and direction of their impacts on these perceptions may vary.

The analysis shows that aesthetic perception is positively correlated with Coastal Openness (CO, r = 0.516) and Color Diversity (CD, r = 0.431), indicating that open coastal views and diverse color palettes significantly enhance aesthetic perception by providing more attractive and enjoyable visual experiences. Open coastal areas give a sense of vastness and connection with nature, while diverse colors increase visual richness and artistic appeal. Conversely, aesthetic perception is negatively correlated with Landscape Complexity (LC, r = -0.327) and Interference Factor Index (IFI, r = -0.427). Excessive landscape complexity may create a visual overload, reducing the harmony of the scene, while interference factors such as clutter or dissonant elements further diminish the overall aesthetic quality.

For distinctiveness perception, Coastal Openness (CO), Marine-related Element Index (MREI), and Blue Visual Index (BVI) all show strong positive correlations, indicating that expansive coastal views, the addition of marine elements, and blue hues significantly enhance the perception of distinctiveness. These factors add unique visual symbols to the landscape, improving the recognizability and memorability of the scene. In contrast, Building Visibility (BV, r = 0.466) is negatively correlated with distinctiveness perception, as an excess of buildings may obstruct natural or distinctive landscape elements, reducing their uniqueness. Additionally, as the Green Visual Index (GVI) increases, the presence of large areas of vegetation in the image attracts human attention, leading to a decrease in distinctiveness perception.

### 3.2 Effectiveness of the perception prediction model

Two regression models, the OLS regression model and the RF model, were used for fitting validation, and the optimal perceptual simulation model was selected based on the comparison of simulation results.

Through Pearson correlation analysis, we identified strong linear relationships between variables and scoring outcomes. However, due to potential interactions among these influencing factors, a stepwise forward method was chosen during the OLS model construction. Aesthetic and distinctiveness perception scores were utilized as dependent variables, with landscape features filtered through correlation analysis serving as independent variables. In this process, we set a significance level of p < 0.05 for the included variables. Stepwise regression was employed to incorporate independent variables into the regression model, progressively excluding unsuitable landscape features to aim for an improved adjusted Coefficient of Determination (R^2^) value. In the final OLS regression model, three feature indicators were selected for aesthetic perception and two for distinctiveness perception, with fitting coefficient information provided in Table 4. Despite our hopes for a higher R^2^ value, the fitting results of the OLS regression model could only reach a maximum of 0.581 and 0.634, highlighting its explanatory limitations. Importantly, even if all factors were included in the linear regression model, the fitting results would not necessarily improve.

**Table 4:** Linear regression model fitting of human perception.

Dependent variables	Model	Unstandardized Coefficients		Standardized Coefficients	t	Sig.
		B	Std. Error	Beta		
Aesthetic perception	(Constant)	4.02	0.185		21.705	0.000
	CO	1.972	0.491	0.463	4.014	0.000
	IFI	−2.157	0.61	−0.404	−3.537	0.001
	CD	0.832	0.284	0.338	2.928	0.006
Distinctiveness perception	(Constant)	3.000	0.239		12.530	0.000
	CO	4.809	0.668	0.717	7.195	0.000
	MREI	121.337	28.998	0.417	4.184	0.000

**Fig 8:**
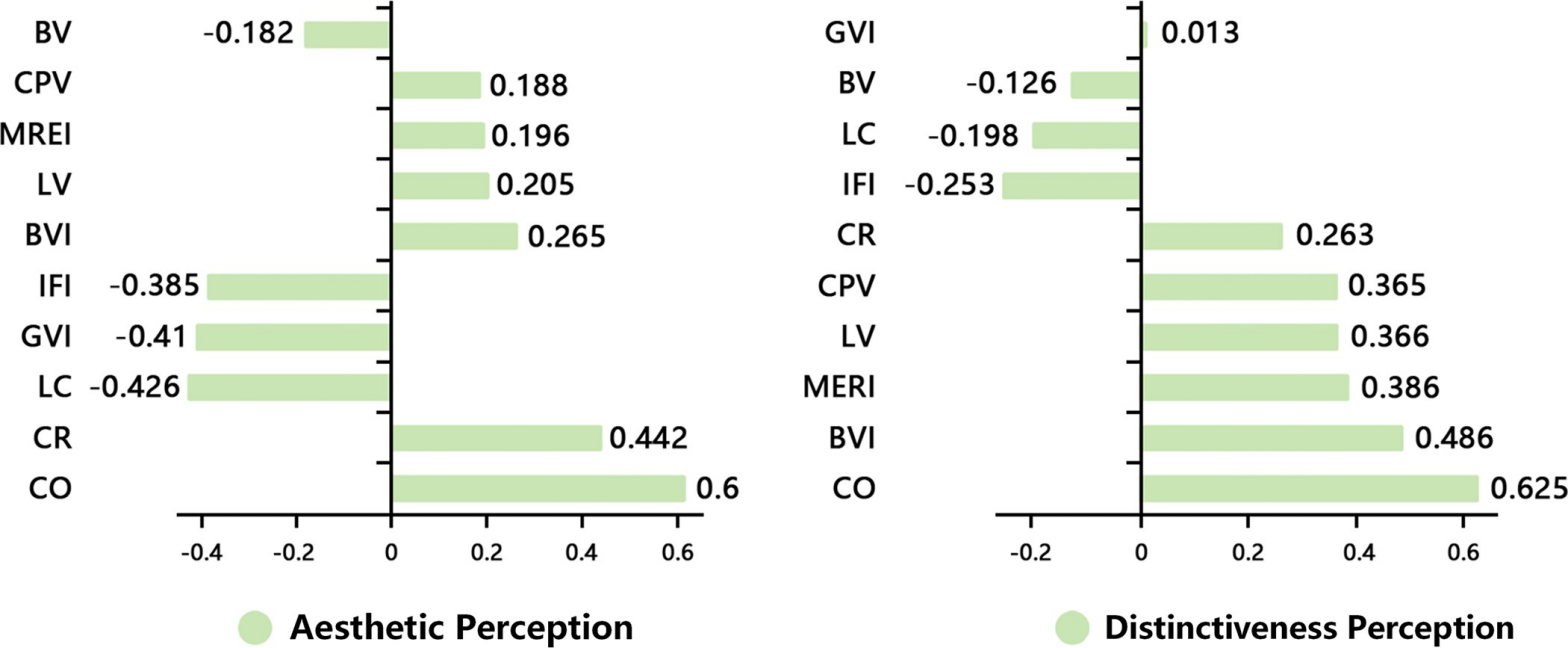
Importance of random forest model.

For the random forest model, when the n-estimators parameter was set to 100, max-depth to 12, max-features to 4, min-samples-leaf to 5, and min-samples-split to 5, the model demonstrated the best accuracy and generalization capability, achieving the highest accuracy rates. We then calculated the R^2^ and Root Mean Squared Error (RMSE) values for both the multiple regression and random forest regression models. At this point, the R^2^ for aesthetic and distinctiveness perception reached 0.868 and 0.773, respectively. From the importance ranking of influencing factors in the random forest model (Fig 8), it can be seen that the importance of the blue visual index and coastal openness is significantly higher than that of other landscape features in terms of distinctiveness perception. For aesthetic perception, the importance of coastal openness is the highest. This result is highly consistent with previous Pearson analysis results, proving the effectiveness of this study.

The comparison of model fittings indicates that the R^2^ of the OLS regression model is significantly lower than that of the random forest model (Table 5). At the same time, the RMSE values of the OLS model are higher than those of the random forest model. The comparison of model fittings indicates that the R^2^ of the OLS regression model is significantly lower than that of the random forest model (Fig 5). Therefore, when predicting visual preference scores based on the proportion of landscape features, the fitting effect and prediction results of the random forest model were superior to those of the OLS regression model.

**Table 5:** Comparison of fitting effects between linear regression and random forest models.

Perceived features	OSL regression model		RF model	
	R^2^	RMSE	R^2^	RMSE
aesthetic perception	0.518	0.398	0.868	0.286
distinctiveness perception	0.634	0.547	0.773	0.305

### 3.3 Coupling analysis of aesthetics with distinctiveness perception

#### 3.3.1 Aesthetics and distinctiveness perception prediction map

**Fig 9:**
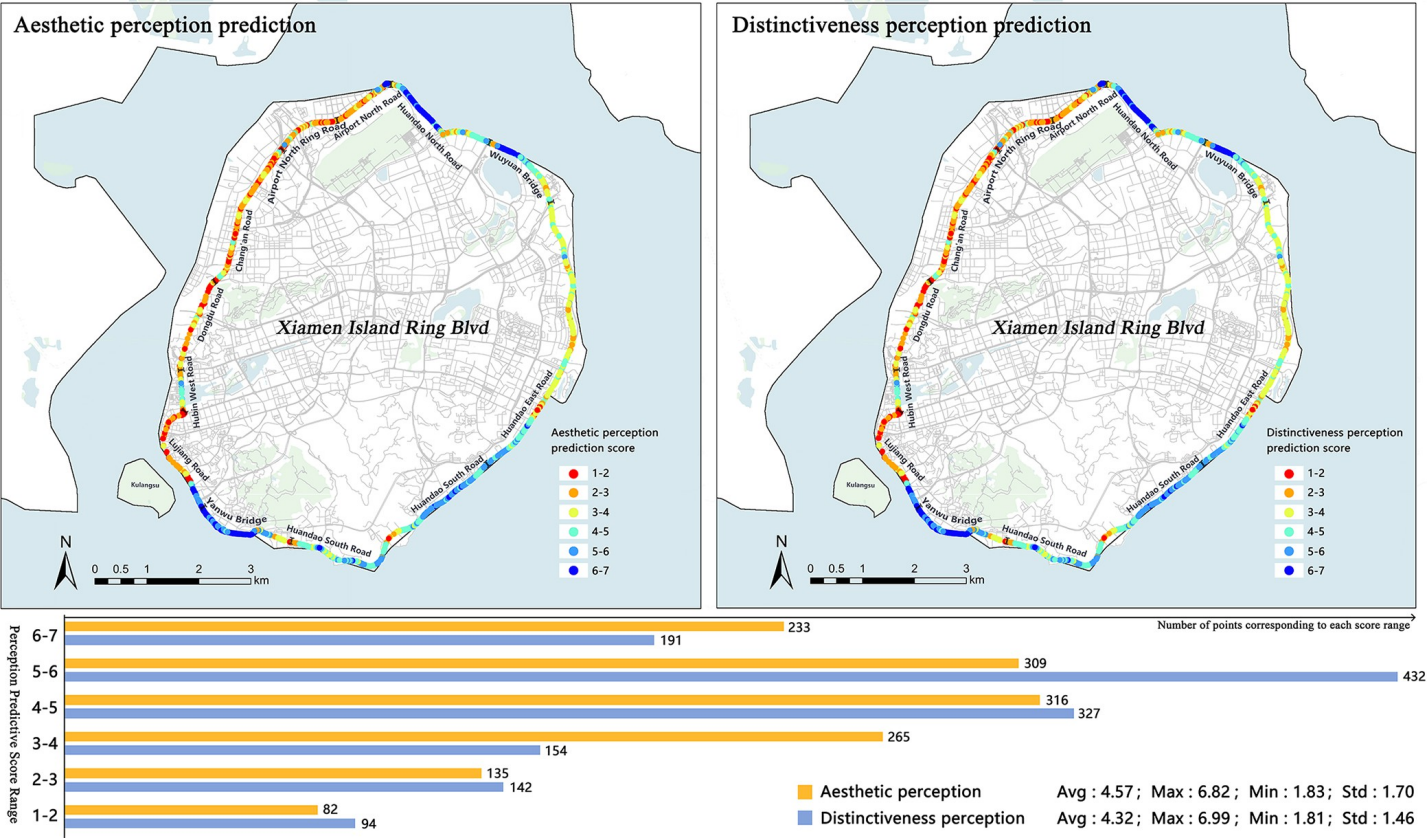
Perception prediction map and numerical grade distribution.

A random forest model was used to predict the visual scoring of the remaining 1492 photos that had not been manually scored. Subsequently, the scores were imported within the study area into ArcGIS for graphical analysis (Fig 9).

The results show that the average predicted score for aesthetic perception in the study area was 4.57 and that for distinctiveness perception was 4.49, both of which were in the upper middle level of the 7-level Likert scale.

The sections with high aesthetic perception are mainly concentrated in the Huandao North Road, Wuyuan Bridge, Yanwu Bridge, and Huandao South Road Yefengzhai sections. The aesthetic perception score of the Dongdu Road to Airport North Road section is low. The SVI of this road section has a low blue visual index, a high green visual index, and a high building visibility index. Thus, the coastal openness is low, the overall landscape changes little, and the color richness is low.

The overall score for distinctiveness perception is high in the east and low in the west, with the higher areas primarily concentrated in the Yanwu Bridge section. The distribution patterns of the different scoring sections were also different. The scenes with high distinctiveness perception scores exhibit a sequential pattern and are concentrated in the Yanwu Bridge, Huandao North Road, and Wuyuan Bridge sections. The scenes with low scores have breakpoint mutations and are mainly in the section from Dongdu Road to the north Ring Road of the airport. Road sections with lower predicted scores are distributed in the western section. The continuity and frequency of the low score of the Chang’an Road section are higher because of the higher green visual index in this section.

#### 3.3.2 Coupling classification of aesthetic and distinctiveness perception

**Fig 10:**
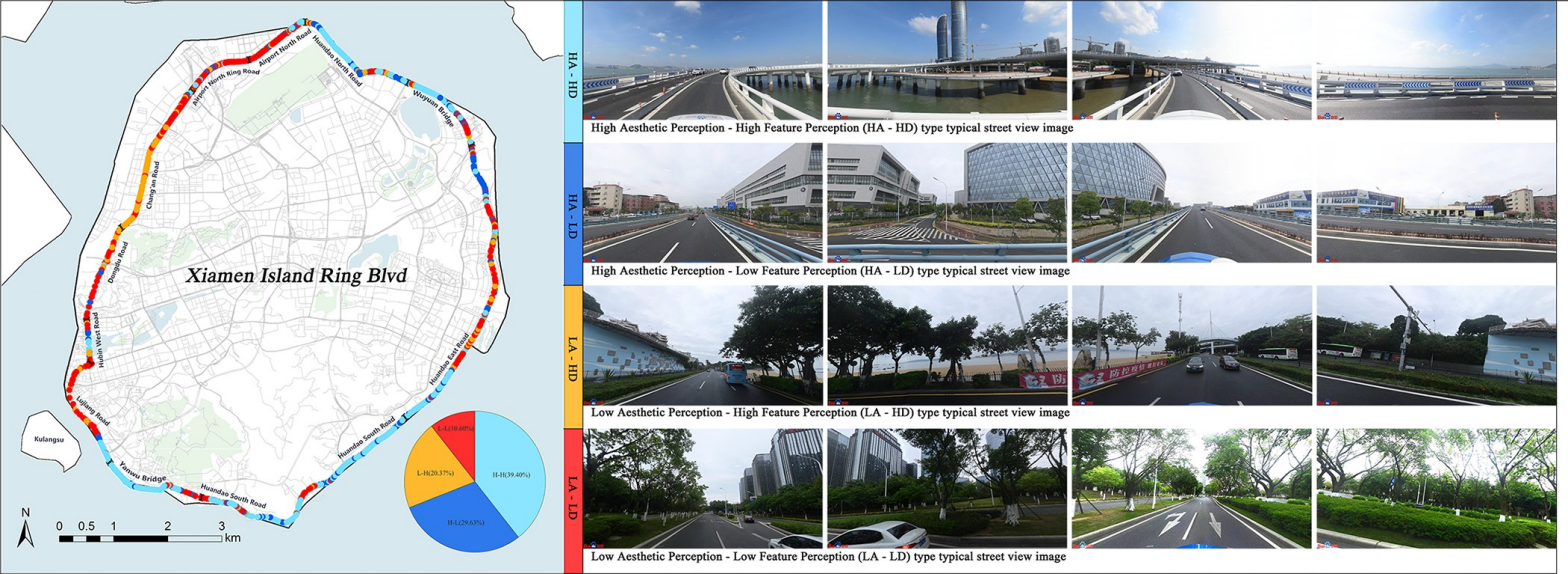
Perception type distribution and typical images.

We coupled aesthetic perception and distinctiveness perception and categorized them into four different types of street segments (Fig 10), including high aesthetic perception and high distinctiveness perception (HA-HD), high aesthetic perception and low distinctiveness perception (HA-LD), low aesthetic perception and high distinctiveness perception (LA-HD) and low aesthetic perception and low distinctiveness perception (LA-LD).

The HA-HD type accounts for 39.4%, mainly located in the northeast and southern sections of the Island Ring Boulevard, where the roads are in closer proximity to the coastline. The HA-LD type is primarily concentrated in the eastern region of the Island Ring Boulevard, and is characterized by a lack of distinctive coastal urban landscape; however, it does possess notable aesthetic value in terms of road scenery. The LA-HD type is concentrated along Chang’an Road, exhibiting coastal urban features such as prominent landmarks and urban furniture Nevertheless, the arrangement of landscape features in this particular road section is disorderly, leading into lower degrees of both formal aesthetics and perceptual aesthetics. The LA-LD type is predominantly located in the vicinity of the Airport North Ring Road and Lujiang Road, accounting for 10.5%. This section of the road has the potential and need for improvement in terms of its visual appeal and distinctive characteristics.

## 4 Discussion

### 4.1 Impact of street view features to aesthetics and distinctiveness perception

The results of the correlation analysis and the importance ranking of influencing factors in the random forest model show that coastal openness and color diversity have the greatest impact on aesthetic perception. Conventional street research has shown that denser plants, buildings, and walls decrease the proportion of the sky, creating a strong sense of enclosure that facilitates the human sense of direction on the street and improves the quality of the street [20,65]. Distinct from typical urban streets, coastal openness significantly affects both aesthetic and distinctiveness perceptions, indicating that the human expectations for coastal roads tend to favor a wide panoramic view rather than an extended perspective. Thus, enhancing sea view visibility and coastal openness involves reducing building density and vegetation around coastal roads.

Our findings suggest that richer color schemes and simpler landscape benefit the human perception of coastal street aesthetics. This finding is similar to previous street landscape research, which concluded that the attractiveness of a street depends on its complexity [66]. Owing to the unique geographical feature of the coastal area, the public’s expectation for the landscape of coastal roads tends to focus more on the embodiment of the coastal style. Urban coastal roads with rich colors and simple landscape elements meet the public’s imagination of what a ‘coastal road’ should be. There is a significant difference between the landscapes of coastal roads and noncoastal areas. Urban coastal roads with a single change in landscape elements facilitate the formation of a continuous spatial imagery. This is because the continuity of the landscape has a significant impact on people’s landscape evaluation [67]. However, road sections with a high landscape interference index may confuse the human view because of excessive elements, which decreases aesthetic perception. Therefore, the human aesthetic perception can be enhanced by reducing complex landscape elements and enriching visual landscape colors.

This study explores how physical features influence human perception of coastal road feature. A high distinctiveness perception index indicates that the road landscape is unique and attractive [68]. Previous studies have shown that landmark buildings and iconic landscape elements can attract human attention and make street views more unique [69]. This argument has been further demonstrated in this study. Previous studies have shown that water has a positive impact on the aesthetic quality of the landscape [70,71]. Marine-related elements are key features that distinguish the urban coastal landscape from that of other cities. The blue visual index is the most intuitive indicator of the marine landscape and can significantly improve the human perception of the distinctiveness of Island Ring Boulevard.

Previous studies have reported the green visual index to be the most important landscape indicator affecting spatial perception, and it typically has a positive impact on the human perception of streets [72]. However, we found no significant correlation between the green visual index and human aesthetic perception in coastal area. In contrast, a higher green visual index reduces the level of human distinctiveness perception. This is because a higher green visual index obstructs the interface between the road and the ocean, thereby reducing the human perception of the distinctiveness of coastal cities.

In contrast to urban mountain view enhancement by controlling building heights, ocean view visibility in cities faces stricter conditions [73]. Open waterfront areas have distinct feature that are easily noticed, remembered, and identified, which can guide the public back to the waterfront. Marine-related elements are key features that distinguish urban coastal landscapes from those of other cities. Therefore, landscape elements related to the ocean can substitute for ocean perception, and improving the visibility of landscape elements related to the ocean can significantly enhance the distinctiveness perception of coastal roads. Landscape elements such as islands, beaches, and ships can positively influence the human perception of coastal scenery, achieving the effect of not seeing the ocean but being able to perceive it. Therefore, in sections with limited blue views, enhancing the visibility of ocean-related elements can be beneficial, thereby improving the human perception of the coastal image. This is consistent with previous studies, which mentioned that trees in a line or strip more than tree in a group in landscape improves the aesthetic quality [74].

### 4.2 The inspiration for enhancing the coastal road environment

Our results reveal that the low perception section of coastal roads accounts for 60.6% of Island Ring Boulevard, highlighting the urgent need for road landscape improvement. Similar phenomena have been observed in some cities in China, such as Wuhan [75]. The common root of such problems lies in the lack of targeted improvement in road landscape. Therefore, we propose suggestions to improve the visual landscape of urban coastal roads on Island Ring Boulevard based on the coupling results of aesthetics and unique perception:

(1) HA-HD type: We recommend maintaining the current state of affairs in areas where there is a high level of both aesthetic appeal and perception of distinctiveness. For example, the southern sections of the Island Ring Boulevard have a high concentration of tourist attractions, which results in relatively higher landscape quality in that region. These areas not only offer expansive views of the coastline but also hold significant symbolic importance as urban landmarks. This category also encompasses regions with a significant blue visual index, where individuals can directly engage with the ocean in close proximity. Simultaneously, the combinations of landscape elements in this area exhibit minimal variation, leading to a consistent and uninterrupted coastal landscape that offers an enjoyable landscape experience.

(2) HA-LD type: We have identified the areas where there is a disparity between the perception of distinctiveness and aesthetics, with distinctiveness being perceived as low and aesthetics being perceived as high. We recommend integrating urban furniture with marine elements along the road to create a captivating and visually appealing road landscape. For road sections with a significant green view index, the proportion of shrubs can be moderately reduced, and an alternating arrangement of roadside trees can be adopted. This method not only opens up the coastal view but also serves as a "framed view" technique in landscape design.

(3) LA-HD type: We suggest streamlining the road landscape elements and enhancing color richness in regions that are highly noticeable but lack aesthetic appeal. Accordingly, our proposal suggests utilizing roadways, water bodies, vegetation, limited artificial structures, and the sky as the primary elements to create the coastal road landscape. This is likely to establish a structured coastal road, which will contribute to the establishment of a consistent image of urban coastal roads. Urban roads serve as conduits for the cultural ambiance of the city, which is commonly manifested through architectural designs and structures [76]. The buildings lining both sides of Island Ring Boulevard can incorporate brick red roofs, while the walls can be predominantly white and warm gray [53]. This color scheme is consistent with local traditional residences and maintains regional style consistency. It also increases the richness of landscape colors, forming a coastal space with harmonious colors.

(4) LA-LD type: Our investigation revealed that regions characterized by both low aesthetic appeal and limited perception of distinctiveness are predominantly enclosed and have obstructed views. If circumstances allow, dismantle the temporary structures located along the coast in order to enhance the coastal openness and the blue visual index, thereby fulfilling the public’s aspiration to admire and approach the ocean.

### 4.3 Research limitations

There are still remainning issue and limitations that warrant futher exploration in the future. One limitation pertains to SVIs. Although big data SVIs partially represent urban road environments, they have limitations. Collected SVIs, mainly from the past, may not fully represent current road environments. In future research, the latest SVI samples will be collected for further analysis. Furthermore, SVIs effectively predict static elements but fail to represent dynamic elements such as motor vehicles and pedestrians. Therefore, they should be further combined with data such as crowd heat maps to improve the accuracy of the study. In addition, the SVI quality of coastal areas, which is impacted by weather during capture, significantly influences human perception.

Research focused on visual perception cannot fully encapsulate human perception. Although human perception is closely related to vision, human perception of a city is related to many factors such as odor, sound, and psychological state. SVIs can only represent the visual part of perception. Moreover, the images of landscape elements only cover some of their feature. For example, even if buildings occupy the same area, differences in architectural style can affect the human perception of them. In future research, identifying, distinguishing, and categorizing the styles and other feature of landscape elements will help improve the accuracy of perception quantification. To further validate the effectiveness of the random forest model in predicting aesthetic and distinctiveness perceptions, we plan to compare it with other advanced machine learning techniques and deep learning architectures in future research. These comparisons will include but not be limited to SVM, gradient boosting trees (GBM), CNN, and recurrent neural networks (RNN) [77].

Finally, the selected objects and samples in this study are subject to certain limitations. The road feature, vegetation types, and urban atmospheres of coastal roads in different cities vary. The regression model, based on Island Ring Boulevard, may not apply universally to all coastal roads. Future research should be expanded to include diverse coastal city road landscapes, creating a comprehensive database to improve research universality. At the same time, we recommend that future research could explore incorporating additional features or data sources, such as seasonal variations or socio-economic factors, to further refine the predictions of human perception [78].

## 5. Conclusions

Urban waterfront areas, which are vital natural resources and highly valued public spaces within cities, play a pivotal role in enhancing the overall urban environment. Our study employed SVIs and utilized deep learning techniques to quantify street view elements and features, thereby simulating human aesthetics and distinctiveness perceptions of urban coastal roads. In addition, we established an innovative approach of urban coastal road perception prediction through Pearson correlation analysis and random forest model. We classified roads based on varying levels of perception and provided targeted suggestions for enhancing road landscapes. Our research not only explores the influence of street view features coupled with aesthetics and distinctiveness perception, but also predicts human perception of urban coastal roads. By effectively adopting different landscape improvement strategies for different road sections, this study offers innovative perspectives and precise strategies for enhancing urban waterfront areas landscapes.

## Supporting information

S1 File (DOCX)

## References

[1] Li X, Wang X, Jiang X, Han J, Wang Z, Wu D, et al. Prediction of riverside greenway landscape aesthetic quality of urban canalized rivers using environmental modeling. Journal of Cleaner Production. 2022;367. doi: 10.1016/j.jclepro.2022.133066 WOS:000830781200003.

[2] Sairinen R, Kumpulainen S. Assessing social impacts in urban waterfront regeneration. Environmental Impact Assessment Review. 2006;26(1):120–35. doi: 10.1016/j.eiar.2005.05.003 WOS:000234454900006.

[3] Yao Y, Zhu X, Xu Y, Yang H, Wu X, Li Y, et al. Assessing the visual quality of green landscaping in rural residential areas: the case of Changzhou, China. Environmental Monitoring and Assessment. 2012;184(2):951–67. doi: 10.1007/s10661-011-2012-z WOS:000298505400023. 21479559

[4] Chen J, Wang X, Wu Y, Shi B, Lu J. Environmental Evaluation of Small Coastal Cities Based on Street View Images. Journal of Physics: Conference Series. 2021;1881(4):042083. doi: 10.1088/1742-6596/1881/4/042083

[5] SU T, LIU W, WU W, CHEN J. Research progress and prospect of urban waterfront redevelopment. 2023;42(2):392–405. 10.18306/dlkxjz.2023.02.015.

[6] Sun D, Li Q, Gao W, Huang G, Tang N, Lyu M, et al. On the relation between visual quality and landscape characteristics: a case study application to the waterfront linear parks in Shenyang, China. Environmental Research Communications. 2021;3(11). doi: 10.1088/2515-7620/ac34c7 WOS:000723864800001.

[7] Kati V, Jari N. Bottom-up thinking Identifying socio-cultural values of ecosystem services in local blue-green infrastructure planning in Helsinki, Finland. Land Use Policy. 2016; 50:537–47. doi: 10.1016/j.landusepol.2015.09.031 WOS:000367755700049.

[8] Pouso S, Borja A, Fleming LE, Gomez-Baggethun E, White MP, Uyarra MC. Contact with blue-green spaces during the COVID-19 pandemic lockdown beneficial for mental health. Science of the Total Environment. 2021;756. doi: 10.1016/j.scitotenv.2020.143984 WOS:000603487500087. 33277006 PMC7688424

[9] Dai L, Zheng C, Dong Z, Yao Y, Wang R, Zhang X, et al. Analyzing the correlation between visual space and residents’ psychology in Wuhan, China using street-view images and deep-learning technique. City and Environment Interactions. 2021; 11:100069. 10.1016/j.cacint.2021.100069.

[10] Gascon M, Sánchez-Benavides G, Dadvand P, Martínez D, Gramunt N, Gotsens X, et al. Long-term exposure to residential green and blue spaces and anxiety and depression in adults: A cross-sectional study. Environmental Research. 2018; 162:231–9. doi: 10.1016/j.envres.2018.01.012 29358115

[11] de Vries S, Verheij RA, Groenewegen PP, Spreeuwenberg P. Natural Environments—Healthy Environments? An Exploratory Analysis of the Relationship between Greenspace and Health. Environment and Planning A: Economy and Space. 2003;35(10):1717–31. doi: 10.1068/a35111

[12] Zhou X, Cen Q, Qiu H. Effects of urban waterfront park landscape elements on visual behavior and public preference: Evidence from eye-tracking experiments. Urban Forestry & Urban Greening. 2023;82. doi: 10.1016/j.ufug.2023.127889 WOS:000951648200001.

[13] Binyi LIU. Towards Landscape Interaction: Inheritance and Development of Landscape Perception and Visual Evaluation. Landscape Architecture. 2022;29(9):12–7. doi: 10.14085/j.fjyl.2022.09.0012.06

[14] Bulut Z, Yilmaz H. Determination of waterscape beauties through visual quality assessment method. Environmental Monitoring and Assessment. 2009;154(1–4):459–68. doi: 10.1007/s10661-008-0412-5 WOS:000267824400040. 18584297

[15] Filova L, Vojar J, Svobodova K, Sklenicka P. The effect of landscape type and landscape elements on public visual preferences: ways to use knowledge in the context of landscape planning. Journal of Environmental Planning and Management. 2015;58(11):2037–55. doi: 10.1080/09640568.2014.973481 WOS:000360142700008.

[16] Ye Y, Zeng W, Shen Q, Zhang X, Lu Y. The visual quality of streets: A human-centred continuous measurement based on machine learning algorithms and street view images. Environment and Planning B-Urban Analytics and City Science. 2019;46(8):1439–57. doi: 10.1177/2399808319828734 WOS:000485946000005.

[17] Tang J, Long Y. Measuring visual quality of street space and its temporal variation: Methodology and its application in the Hutong area in Beijing. Landscape and Urban Planning. 2019;191. doi: 10.1016/j.landurbplan.2018.09.015 WOS:000491614300008.

[18] Wu B, Yu B, Shu S, Liang H, Zhao Y, Wu J. Mapping fine-scale visual quality distribution inside urban streets using mobile LiDAR data. Building and Environment. 2021; 206:108323. 10.1016/j.buildenv.2021.108323.

[19] Yun HY, Zegras C, Palencia Arreola DH. “Digitalizing Walkability”: Comparing Smartphone-Based and Web-Based Approaches to Measuring Neighborhood Walkability in Singapore. Journal of Urban Technology. 2019;26(3):3–43. doi: 10.1080/10630732.2019.1625016

[20] Harvey C, Aultman-Hall L, Hurley SE, Troy A. Effects of skeletal streetscape design on perceived safety. Landscape and Urban Planning. 2015; 142:18–28. doi: 10.1016/j.landurbplan.2015.05.007 WOS:000363350100003.

[21] Qiu W, Li W, Liu X, Huang X. Subjectively Measured Streetscape Perceptions to Inform Urban Design Strategies for Shanghai. Isprs International Journal of Geo-Information. 2021;10(8). doi: 10.3390/ijgi10080493 WOS:000689072900001.

[22] Li Z, Sun X, Zhao S, Zuo H. Integrating eye-movement analysis and the semantic differential method to analyze the visual effect of a traditional commercial block in Hefei, China. Frontiers of Architectural Research. 2021;10(2):317–31. 10.1016/j.foar.2021.01.002.

[23] Nagata S, Nakaya T, Hanibuchi T, Amagasa S, Kikuchi H, Inoue S. Objective scoring of streetscape walkability related to leisure walking: Statistical modeling approach with semantic segmentation of Google Street View images. Health & Place. 2020;66. doi: 10.1016/j.healthplace.2020.102428 WOS:000594147200001. 32977303

[24] Ewing R, Handy S. Measuring the Unmeasurable: Urban Design Qualities Related to Walkability. Journal of Urban Design. 2009;14(1):65–84. doi: 10.1080/13574800802451155

[25] Hamidi S, Moazzeni S. Examining the Relationship between Urban Design Qualities and Walking Behavior: Empirical Evidence from Dallas, TX. 2019;11(10):2720. doi: 10.3390/su11102720

[26] Misthos L-M, Krassanakis V, Merlemis N, Kesidis AL. Modeling the Visual Landscape: A Review on Approaches, Methods and Techniques. Sensors. 2023;23(19). doi: 10.3390/s23198135 WOS:001097411900001. 37836966 PMC10574952

[27] Polednikova Z, Galia T. Photo simulation of a river restoration: Relationships between public perception and ecosystem services. River Research and Applications. 2021;37(1):44–53. doi: 10.1002/rra.3738 WOS:000577469500001.

[28] Ding J, Luo L, Shen X, Xu Y. Influence of built environment and user experience on the waterfront vitality of historical urban areas: A case study of the Qinhuai River in Nanjing, China. Frontiers of Architectural Research. 2023;12(5):820–36. 10.1016/j.foar.2023.05.004.

[29] Zhao X, Lu Y, Lin G. An integrated deep learning approach for assessing the visual qualities of built environments utilizing street view images. Engineering Applications of Artificial Intelligence. 2024;130. doi: 10.1016/j.engappai.2023.107805 WOS:001150068400001.

[30] Zhang F, Wu L, Zhu D, Liu Y. Social sensing from street-level imagery: A case study in learning spatio-temporal urban mobility patterns. Isprs Journal of Photogrammetry and Remote Sensing. 2019; 153:48–58. doi: 10.1016/j.isprsjprs.2019.04.017 WOS:000472590400004.

[31] Li Y, Yabuki N, Fukuda T. Measuring visual walkability perception using panoramic street view images, virtual reality, and deep learning. Sustainable Cities and Society. 2022;86. doi: 10.1016/j.scs.2022.104140 WOS:000852691200001.

[32] Zhang L, Wang L, Wu J, Li P, Dong J, Wang T. Decoding urban green spaces: Deep learning and google street view measure greening structures. Urban Forestry & Urban Greening. 2023;87. doi: 10.1016/j.ufug.2023.128028 WOS:001063619600001.

[33] Pouyanfar S, Sadiq S, Yan Y, Tian H, Tao Y, Reyes MP, et al. A Survey on Deep Learning: Algorithms, Techniques, and Applications. 2018;51(5%J ACM Comput. Surv.):Article 92. doi: 10.1145/3234150

[34] Aboufazeli S, Jahani A, Farahpour M. A method for aesthetic quality modelling of the form of plants and water in the urban parks landscapes: An artificial neural network approach. MethodsX. 2021; 8:101489. doi: 10.1016/j.mex.2021.101489 34434886 PMC8374717

[35] Li Y, Yabuki N, Fukuda T. Exploring the association between street built environment and street vitality using deep learning methods. Sustainable Cities and Society. 2022;79. doi: 10.1016/j.scs.2021.103656 WOS:000781347900002.

[36] Minaee S, Boykov Y, Porikli F, Plaza A, Kehtarnavaz N, Terzopoulos D. Image Segmentation Using Deep Learning: A Survey. IEEE Transactions on Pattern Analysis and Machine Intelligence. 2022;44(7):3523–42. doi: 10.1109/TPAMI.2021.3059968 33596172

[37] Sun D, Ji X, Gao W, Zhou F, Yu Y, Meng Y, et al. The Relation between Green Visual Index and Visual Comfort in Qingdao Coastal Streets. Buildings. 2023;13(2). doi: 10.3390/buildings13020457 WOS:000938306300001.

[38] Luo J, Zhao T, Cao L, Biljecki F. Water View Imagery: Perception and evaluation of urban waterscapes worldwide. Ecological Indicators. 2022;145. doi: 10.1016/j.ecolind.2022.109615 WOS:000886219400003.

[39] Liu Y, Chen M, Wang M, Huang J, Thomas F, Rahimi K, et al. An interpretable machine learning framework for measuring urban perceptions from panoramic street view images. Iscience. 2023;26(3). doi: 10.1016/j.isci.2023.106132 WOS:000995405300001. 36843850 PMC9950426

[40] Cai Q, Abdel-Aty M, Zheng O, Wu Y. Applying machine learning and google street view to explore effects of drivers’ visual environment on traffic safety. Transportation Research Part C-Emerging Technologies. 2022;135. doi: 10.1016/j.trc.2021.103541 WOS:000779699000004.

[41] Clay GR, Daniel TC. Scenic landscape assessment: the effects of land management jurisdiction on public perception of scenic beauty. Landscape and Urban Planning. 2000;49(1–2):1–13. doi: 10.1016/s0169-2046(00)00055-4 WOS:000087062500001.

[42] Gao T, Liang H, Chen Y, Qiu L. Comparisons of Landscape Preferences through Three Different Perceptual Approaches. International Journal of Environmental Research and Public Health. 2019;16(23). doi: 10.3390/ijerph16234754 WOS:000507275700178. 31783624 PMC6926958

[43] Kalivoda O, Vojar J, Skrivanova Z, Zahradnik D. Consensus in landscape preference judgments: The effects of landscape visual aesthetic quality and respondents’ characteristics. Journal of Environmental Management. 2014; 137:36–44. doi: 10.1016/j.jenvman.2014.02.009 WOS:000336359100005. 24594757

[44] Zhang X, Xiong X, Chi M, Yang S, Liu L. Research on visual quality assessment and landscape elements influence mechanism of rural greenways. Ecological Indicators. 2024; 160:111844. 10.1016/j.ecolind.2024.111844.

[45] de la Fuente de Val G, Atauri JA, de Lucio JV. Relationship between landscape visual attributes and spatial pattern indices: A test study in Mediterranean-climate landscapes. Landscape and Urban Planning. 2006;77(4):393–407. doi: 10.1016/j.landurbplan.2005.05.003 WOS:000240085900005.

[46] Chien Y-MC, Carver S, Comber A. An Exploratory Analysis of Expert and Nonexpert-Based Land-Scape Aesthetics Evaluations: A Case Study from Wales. Land. 2021;10(2). doi: 10.3390/land10020192 WOS:000622684200001.

[47] Hauser D, Leopold A, Egger R, Ganewita H, Herrgessell L. Aesthetic perception analysis of destination pictures using #beautifuldestinations on Instagram. Journal of Destination Marketing & Management. 2022;24. doi: 10.1016/j.jdmm.2022.100702 WOS:000798058400002.

[48] Ragheb A, El-Ashmawy R. Urban Waterfront Development for Designing Space in Coastal Cities. International Journal of Sustainable Development and Planning. 2020;15(3):345–52. doi: 10.18280/ijsdp.150311

[49] López-Contreras C, Collantes-Chávez-Costa AL, Barrasa-García S. Indicadores visuales como predictores de la preferencia del paisaje costero en isla Cozumel, México. CienciaUAT. 2022;17(1):35–48. doi: 10.29059/cienciauat.v17i1.1631 SCIELO:S2007-78582022000200035.

[50] Ruowen S, Dan C, Yonghua M, Leilei Q, Ji W. Research on Synergistic Mechanism Between the Protection of Featured Landscape and the Utilization of Stock Space Resources——A Case Study of Chongqing, a Typical Mountainous City. Chongqing Architecture. 2022;21(S1):147–53. doi: 10.3969/j.issn.1671-9107.2022.S1.147

[51] Peng X, Bao Y, Huang Z. Perceiving Beijing’s "City Image" Across Different Groups Based on Geotagged Social Media Data. Ieee Access. 2020; 8:93868–81. doi: 10.1109/access.2020.2995066 WOS:000541123400004.

[52] Ding J, Tao Z, Hou M, Chen D, Wang L. A Comparative Study of Perceptions of Destination Image Based on Content Mining: Fengjing Ancient Town and Zhaojialou Ancient Town as Examples. Land. 2023;12(10). doi: 10.3390/land12101954 WOS:001095139600001.

[53] Miaoyi L, Zhonghao Y, Feng X. Urban Street Greenery Quality Measurement, Planning and Design Promotion Strategies Based on Multi-Source Data: A Case Study of Fuzhou’s Main Urban Area. Landscape Architecture. 2021;28(2):62–8.

[54] Dijkstra N, Bosch SE, van Gerven MAJ. Shared Neural Mechanisms of Visual Perception and Imagery. Trends in Cognitive Sciences. 2019;23(5):423–34. doi: 10.1016/j.tics.2019.02.004 WOS:000467043200009. 30876729

[55] Qiu Y, Qiu Q. Method of Experimental Designand its Applications on Chemical Engineering. Jiangxi Chemical Industry. 2003;(04):60–3.

[56] Huang T, Zhou S, Chen X, Lin Z, Gan F. Colour Preference and Healing in Digital Roaming Landscape: A Case Study of Mental Subhealth Populations. International Journal of Environmental Research and Public Health. 2022;19(17). doi: 10.3390/ijerph191710986 WOS:000851110200001. 36078692 PMC9518100

[57] He Z, Wang Z, Xie Z, Wu L, Chen Z. Multiscale analysis of the influence of street built environment on crime occurrence using street-view images. Computers Environment and Urban Systems. 2022;97. doi: 10.1016/j.compenvurbsys.2022.101865 WOS:000890659200001.

[58] Jiang J, Li F, Yang J, Kang Z, Li J. Construction of indoor obstacle element map based on scene-aware priori obstacle rules. Isprs Journal of Photogrammetry and Remote Sensing. 2022; 195:43–64. doi: 10.1016/j.isprsjprs.2022.11.003 WOS:000889676900001.

[59] Thackway W, Ng M, Lee C-L, Pettit C. Implementing a deep-learning model using Google street view to combine social and physical indicators of gentrification. Computers Environment and Urban Systems. 2023;102. doi: 10.1016/j.compenvurbsys.2023.101970 WOS:001009620800001.

[60] Zhao H, Shi J Qi X, Wang X, Jia J, Ieee, editors. Pyramid Scene Parsing Network. 30th IEEE/CVF Conference on Computer Vision and Pattern Recognition (CVPR); 2017 2017 Jul 21–26; Honolulu, HI2017.

[61] Binyi L, Rong F. Quantitative analysis of the visual attraction elements of landscape space. Journal of Nanjing Forestry University(Natural Sciences Edition). 2014;38(04):149–52.

[62] Norman G. Likert scales, levels of measurement and the "laws" of statistics. Advances in Health Sciences Education. 2010;15(5):625–32. doi: 10.1007/s10459-010-9222-y WOS:000284839900002. 20146096

[63] Svetnik V, Liaw A, Tong C, Culberson JC, Sheridan RP, Feuston BP. Random forest: A classification and regression tool for compound classification and QSAR modeling. Journal of Chemical Information and Computer Sciences. 2003;43(6):1947–58. doi: 10.1021/ci034160g WOS:000186848700029. 14632445

[64] Prasad AM, Iverson LR, Liaw A. Newer Classification and Regression Tree Techniques: Bagging and Random Forests for Ecological Prediction. Ecosystems. 2006;9(2):181–99. doi: 10.1007/s10021-005-0054-1

[65] Ma X, Ma C, Wu C, Xi Y, Yang R, Peng N, et al. Measuring human perceptions of streetscapes to better inform urban renewal: A perspective of scene semantic parsing. Cities. 2021;110. doi: 10.1016/j.cities.2020.103086 WOS:000618543800003.

[66] Spears S. Pedestrian- and Transit-Oriented Design. Journal of Planning Education and Research. 2017;37(4):505–6. doi: 10.1177/0739456x16675469 WOS:000413906900018.

[67] Kuper R. Evaluations of landscape preference, complexity, and coherence for designed digital landscape models. Landscape and Urban Planning. 2017; 157:407–21. 10.1016/j.landurbplan.2016.09.002.

[68] Quercia D, O’Hare NK, Cramer H. Aesthetic capital: what makes london look beautiful, quiet, and happy? 2014.

[69] Ewing R, Handy S, Brownson RC, Clemente O, Winston E. Identifying and Measuring Urban Design Qualities Related to Walkability. Journal of Physical Activity and Health. 2006;3(s1):S223–S40. doi: 10.1123/jpah.3.s1.s223 28834514

[70] Arriaza M, Cañas-Ortega JF, Cañas-Madueño JA, Ruiz-Aviles P. Assessing the visual quality of rural landscapes. Landscape and Urban Planning. 2004;69(1):115–25. 10.1016/j.landurbplan.2003.10.029.

[71] Thiele J, von Haaren C, Albert C. Are river landscapes outstanding in providing cultural ecosystem services? An indicator-based exploration in Germany. Ecological Indicators. 2019; 101:31–40. 10.1016/j.ecolind.2019.01.003.

[72] Lei-qing XU, Ruo-xi M, Zheng C. Fascinating Streets: The Impact of Building Facades and Green View. Landscape Architecture. 2017;- 24(- 10):27–33. doi: 10.14085/j.fjyl.2017.10.0027.07

[73] Lee I, 김충식. Development of an Analysis Method of Visibility Ratio for Urban Landscape Management. Journal of The Urban Design Insitute of Korea. 2002;9(4):23–34. KJD:ART000980720.

[74] Aboufazeli S, Jahani A, Farahpour M. Aesthetic quality modeling of the form of natural elements in the environment of urban parks. Evolutionary Intelligence. 2024;17(1):327–38. doi: 10.1007/s12065-022-00768-1

[75] Zhang X, Xu D, Zhang N. Research on Landscape Perception and Visual Attributes Based on Social Media Data-A Case Study on Wuhan University. Applied Sciences-Basel. 2022;12(16). doi: 10.3390/app12168346 WOS:000846364800001.

[76] Chi S, Xiao H. The Design of Urban Road Landscape under Impacts of Regional Culture: Taking Shenyang Road in Shenyang City as an Example. Journal of Shenyang Jianzhu University (Social Science). 2018;20(04):325–30.

[77] Jahani A, Saffariha M, Barzegar P. Landscape aesthetic quality assessment of forest lands: an application of machine learning approach. Soft Computing. 2023;27(10):6671–86. doi: 10.1007/s00500-022-07642-3

[78] Jahani A. Aesthetic quality evaluation modeling of forest landscape using artificial neural network %J Journal of Wood and Forest Science and Technology. 2017;24(3):17–34. doi: 10.22069/jwfst.2017.11235.1590

