# Supplementary material for: Enhancing the visual environment of urban coastal roads through deep learning analysis of street-view images: A perspective of aesthetic and distinctiveness
Source: PLoS One. 2025 Jan 14;20(1):e0317585. doi: 10.1371/journal.pone.0317585 (PMC11731764; doi:10.1371/journal.pone.0317585)
Supplement: S1 File — (DOCX) [file pone.0317585.s001.docx]

**Aesthetic and Distinctiveness Perception Evaluation of Urban Coastal Roads**

Dear Sir/Madam,

Thank you for participating in this survey on the visual perception of coastal roads on Xiamen Island. The questionnaire consists of 38 sets of images. Please view each image and score them on the following two dimensions. 7 points indicate the highest degree, while 1 point indicates the lowest degree.

This survey is strictly for academic research and is conducted anonymously. Your responses will be kept confidential. Thank you very much for your participation!

1. Please rate this set of images

| 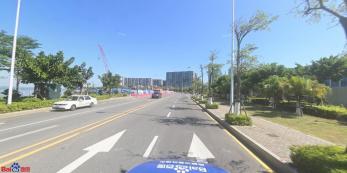 | 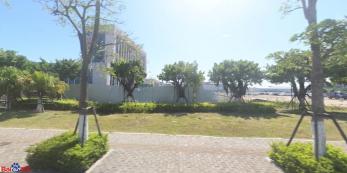 | 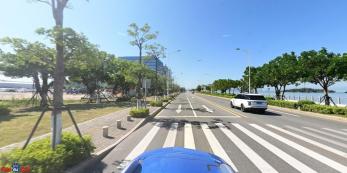 | 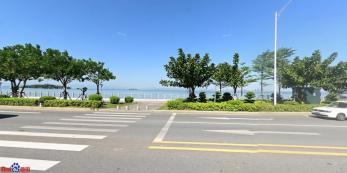 |
| --- | --- | --- | --- |

| Aesthetic Perception: Please rate the beauty of this set of images. |
| --- |
| 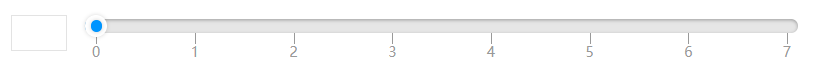 |
| Distinctiveness Perception: Please rate the level of coastal uniqueness for this set of images. |
| 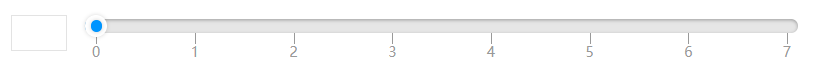 |

1. Please rate this set of images

| 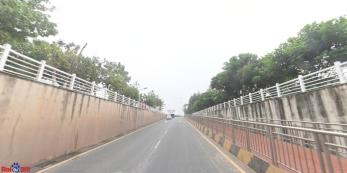 | 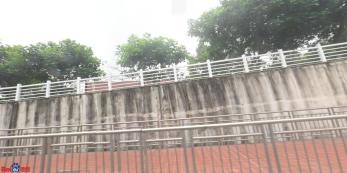 | 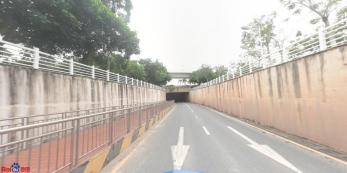 | 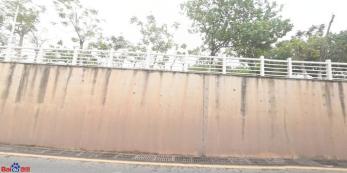 |
| --- | --- | --- | --- |

| Aesthetic Perception: Please rate the beauty of this set of images. |
| --- |
| 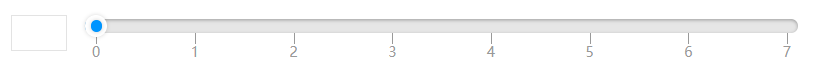 |
| Distinctiveness Perception: Please rate the level of coastal uniqueness for this set of images. |
| 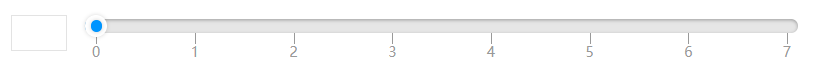 |

1. Please rate this set of images

| 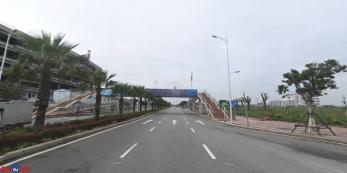 | 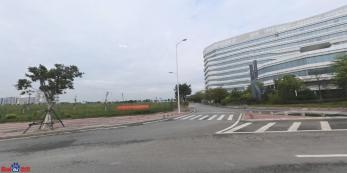 | 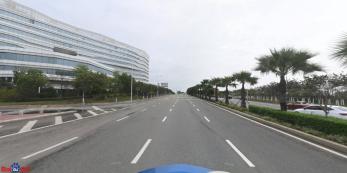 | 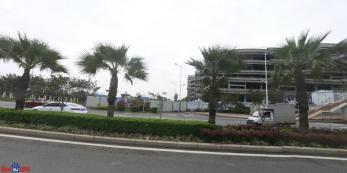 |
| --- | --- | --- | --- |

| Aesthetic Perception: Please rate the beauty of this set of images. |
| --- |
| 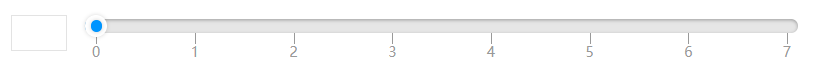 |
| Distinctiveness Perception: Please rate the level of coastal uniqueness for this set of images. |
| 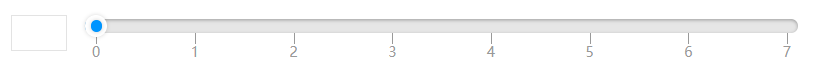 |

1. Please rate this set of images

| 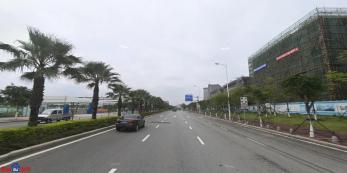 | 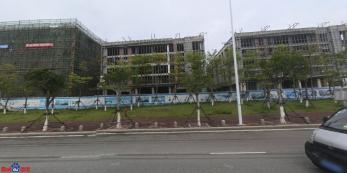 | 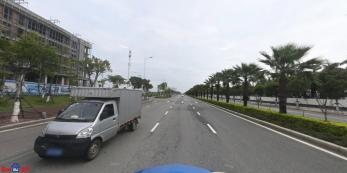 | 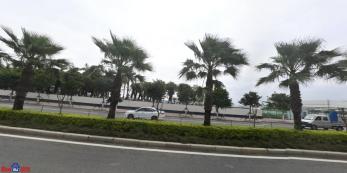 |
| --- | --- | --- | --- |

| Aesthetic Perception: Please rate the beauty of this set of images. |
| --- |
| 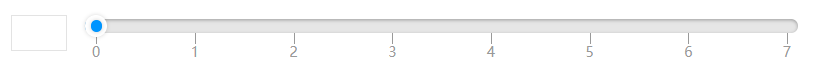 |
| Distinctiveness Perception: Please rate the level of coastal uniqueness for this set of images. |
| 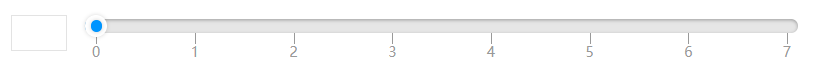 |

1. Please rate this set of images

| 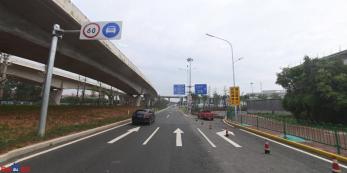 | 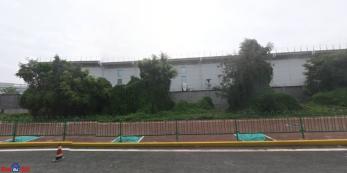 | 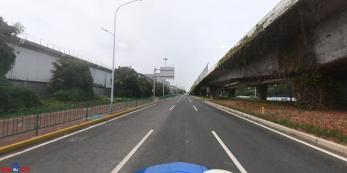 | 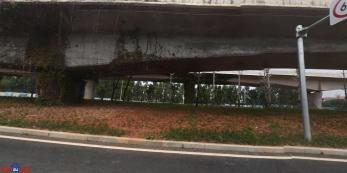 |
| --- | --- | --- | --- |

| Aesthetic Perception: Please rate the beauty of this set of images. |
| --- |
| 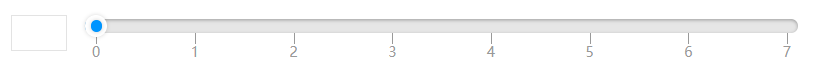 |
| Distinctiveness Perception: Please rate the level of coastal uniqueness for this set of images. |
| 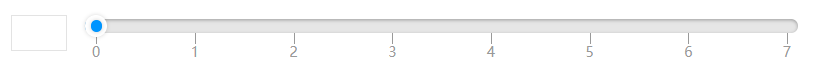 |

1. Please rate this set of images

| 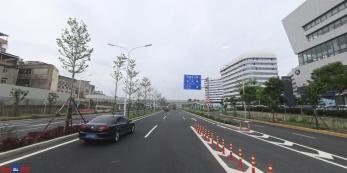 | 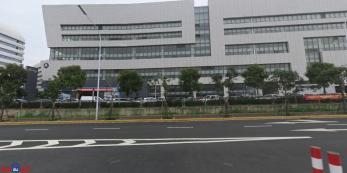 | 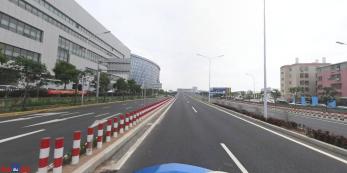 | 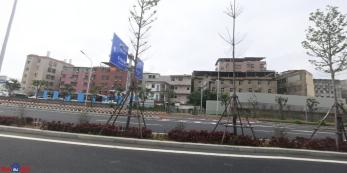 |
| --- | --- | --- | --- |

| Aesthetic Perception: Please rate the beauty of this set of images. |
| --- |
| 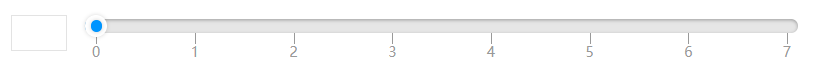 |
| Distinctiveness Perception: Please rate the level of coastal uniqueness for this set of images. |
| 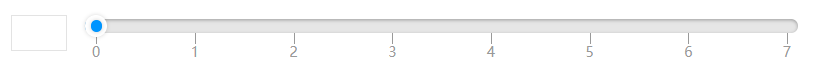 |

1. Please rate this set of images

| 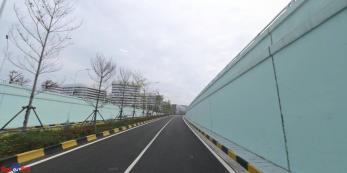 | 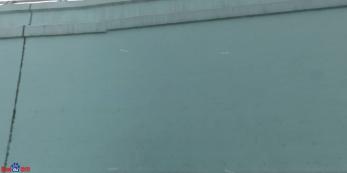 | 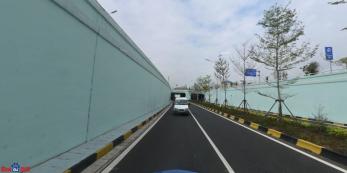 | 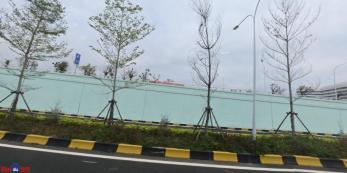 |
| --- | --- | --- | --- |

| Aesthetic Perception: Please rate the beauty of this set of images. |
| --- |
| 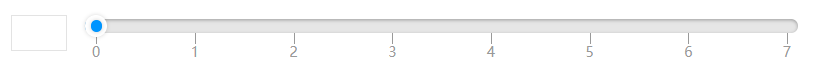 |
| Distinctiveness Perception: Please rate the level of coastal uniqueness for this set of images. |
| 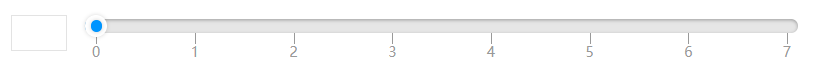 |

1. Please rate this set of images

| 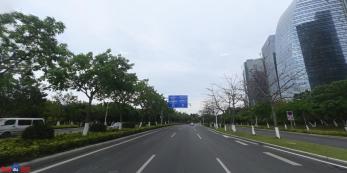 | 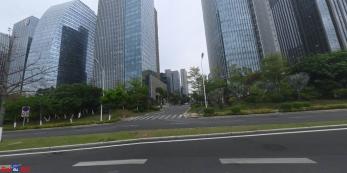 | 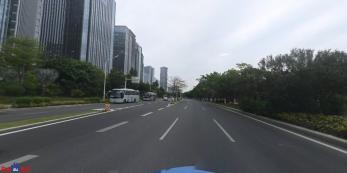 | 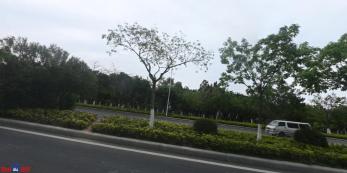 |
| --- | --- | --- | --- |

| Aesthetic Perception: Please rate the beauty of this set of images. |
| --- |
| 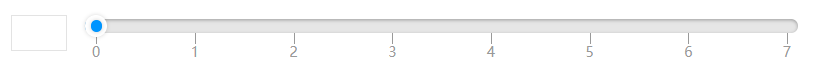 |
| Distinctiveness Perception: Please rate the level of coastal uniqueness for this set of images. |
| 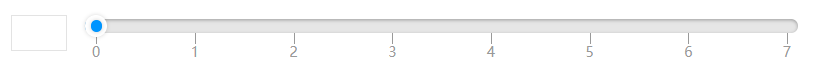 |

1. Please rate this set of images

| 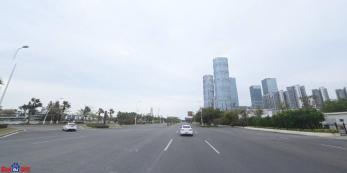 | 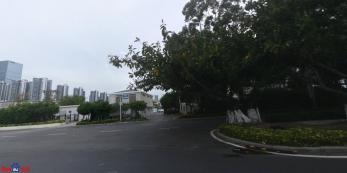 | 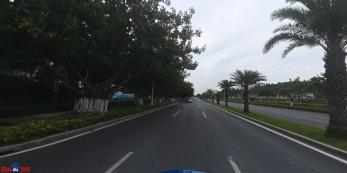 | 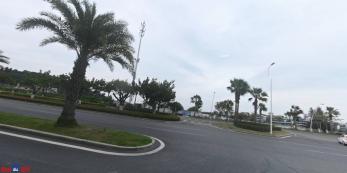 |
| --- | --- | --- | --- |

| Aesthetic Perception: Please rate the beauty of this set of images. |
| --- |
| 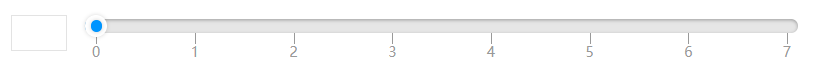 |
| Distinctiveness Perception: Please rate the level of coastal uniqueness for this set of images. |
| 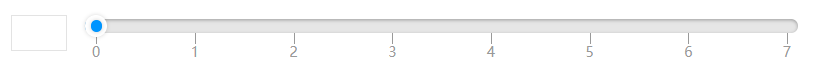 |

1. Please rate this set of images

| 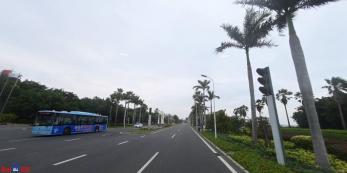 | 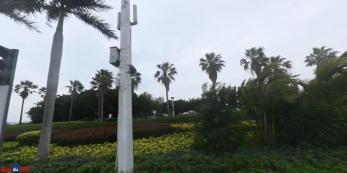 | 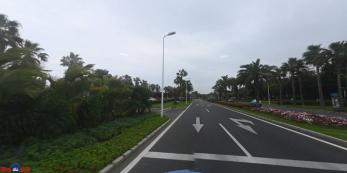 | 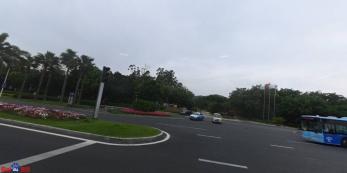 |
| --- | --- | --- | --- |

| Aesthetic Perception: Please rate the beauty of this set of images. |
| --- |
| 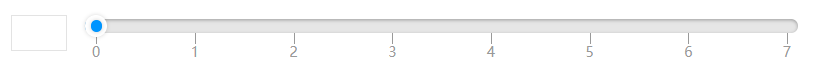 |
| Distinctiveness Perception: Please rate the level of coastal uniqueness for this set of images. |
| 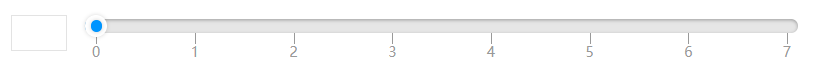 |

1. Please rate this set of images

| 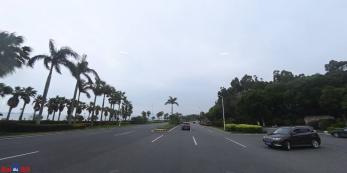 | 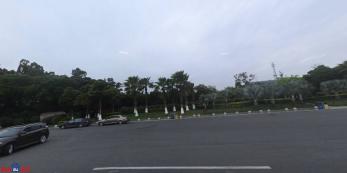 | 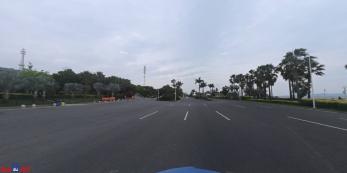 | 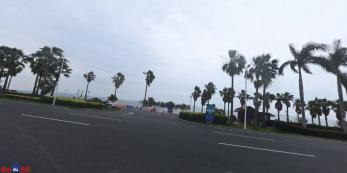 |
| --- | --- | --- | --- |

| Aesthetic Perception: Please rate the beauty of this set of images. |
| --- |
| 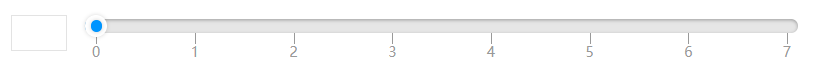 |
| Distinctiveness Perception: Please rate the level of coastal uniqueness for this set of images. |
| 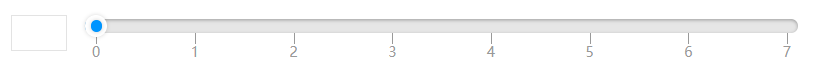 |

1. Please rate this set of images

| 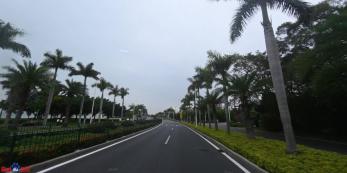 | 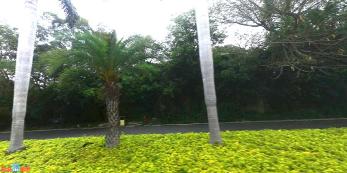 | 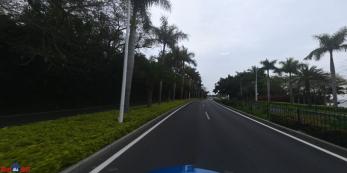 | 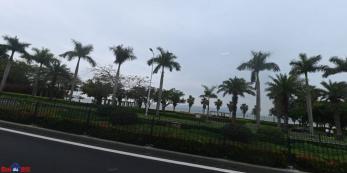 |
| --- | --- | --- | --- |

| Aesthetic Perception: Please rate the beauty of this set of images. |
| --- |
| 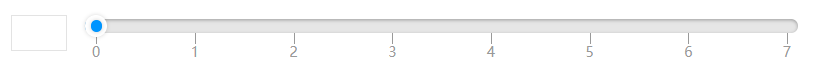 |
| Distinctiveness Perception: Please rate the level of coastal uniqueness for this set of images. |
| 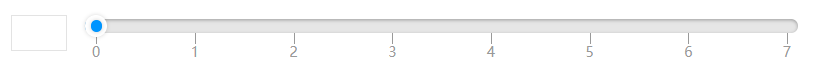 |

1. Please rate this set of images

| 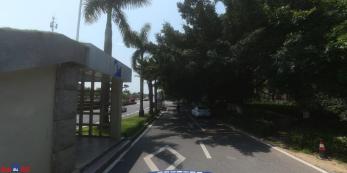 | 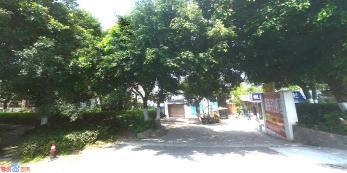 | 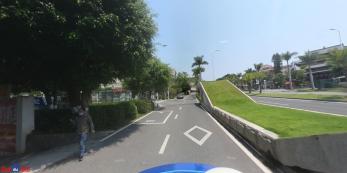 | 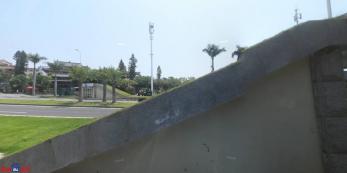 |
| --- | --- | --- | --- |

| Aesthetic Perception: Please rate the beauty of this set of images. |
| --- |
| 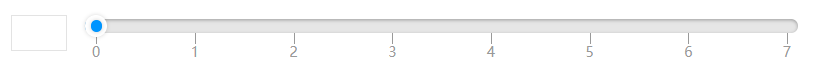 |
| Distinctiveness Perception: Please rate the level of coastal uniqueness for this set of images. |
| 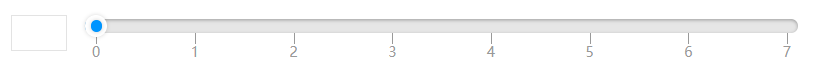 |

1. Please rate this set of images

| 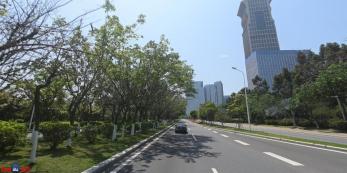 | 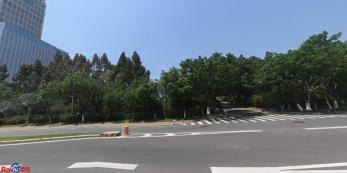 | 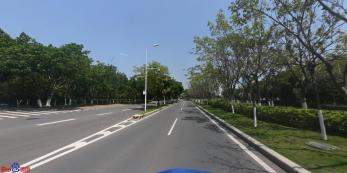 | 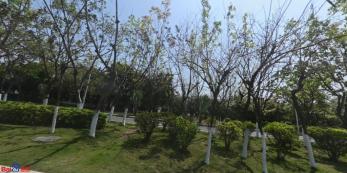 |
| --- | --- | --- | --- |

| Aesthetic Perception: Please rate the beauty of this set of images. |
| --- |
| 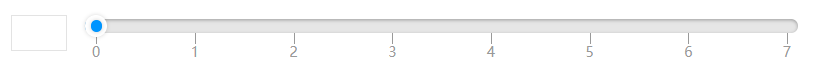 |
| Distinctiveness Perception: Please rate the level of coastal uniqueness for this set of images. |
| 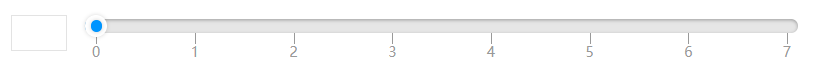 |

1. Please rate this set of images

| 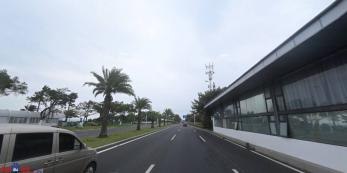 | 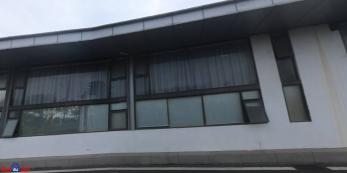 | 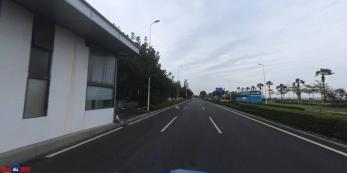 | 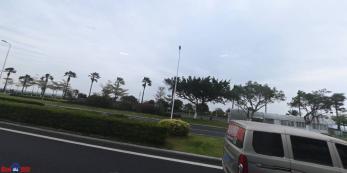 |
| --- | --- | --- | --- |

| Aesthetic Perception: Please rate the beauty of this set of images. |
| --- |
| 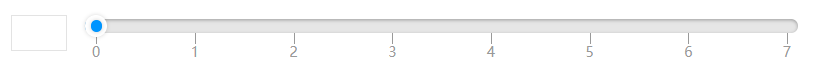 |
| Distinctiveness Perception: Please rate the level of coastal uniqueness for this set of images. |
| 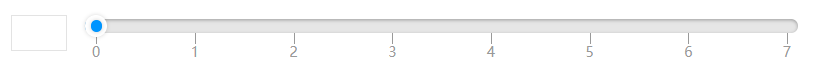 |

1. Please rate this set of images

| 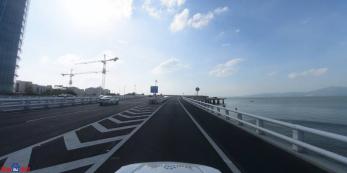 | 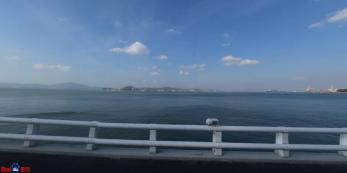 | 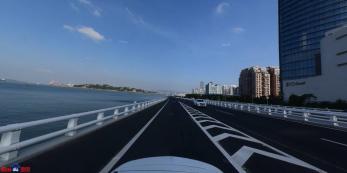 | 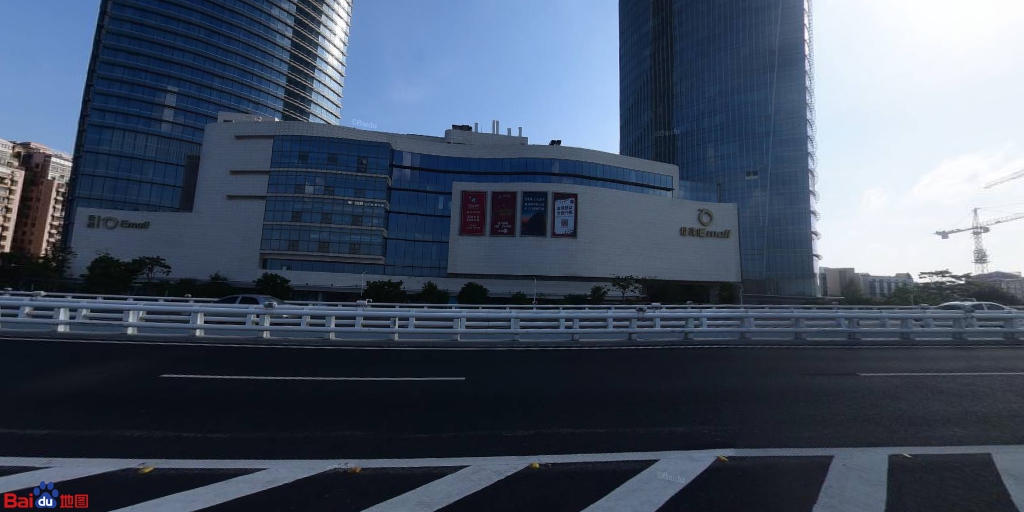 |
| --- | --- | --- | --- |

| Aesthetic Perception: Please rate the beauty of this set of images. |
| --- |
| 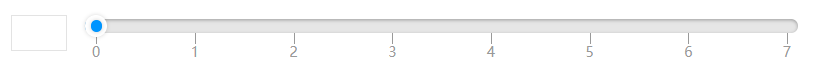 |
| Distinctiveness Perception: Please rate the level of coastal uniqueness for this set of images. |
| 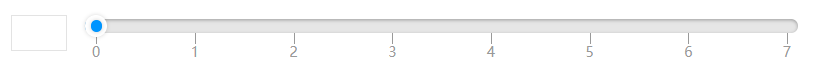 |

1. Please rate this set of images

| 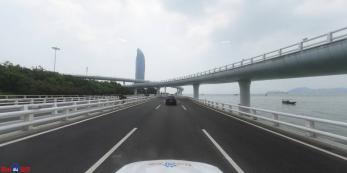 | 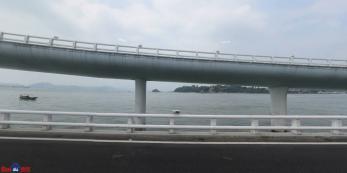 | 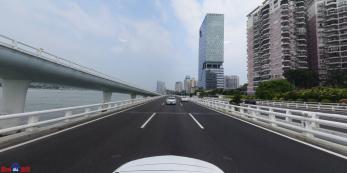 | 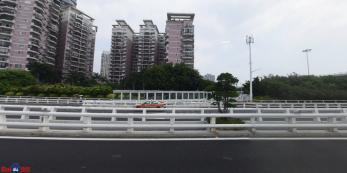 |
| --- | --- | --- | --- |

| Aesthetic Perception: Please rate the beauty of this set of images. |
| --- |
|  |
| Distinctiveness Perception: Please rate the level of coastal uniqueness for this set of images. |
|  |

1. Please rate this set of images

|  |  |  |  |
| --- | --- | --- | --- |

| Aesthetic Perception: Please rate the beauty of this set of images. |
| --- |
|  |
| Distinctiveness Perception: Please rate the level of coastal uniqueness for this set of images. |
|  |

1. Please rate this set of images

|  |  |  |  |
| --- | --- | --- | --- |

| Aesthetic Perception: Please rate the beauty of this set of images. |
| --- |
|  |
| Distinctiveness Perception: Please rate the level of coastal uniqueness for this set of images. |
|  |

1. Please rate this set of images

|  |  |  |  |
| --- | --- | --- | --- |

| Aesthetic Perception: Please rate the beauty of this set of images. |
| --- |
|  |
| Distinctiveness Perception: Please rate the level of coastal uniqueness for this set of images. |
|  |

1. Please rate this set of images

|  |  |  |  |
| --- | --- | --- | --- |

| Aesthetic Perception: Please rate the beauty of this set of images. |
| --- |
|  |
| Distinctiveness Perception: Please rate the level of coastal uniqueness for this set of images. |
|  |

1. Please rate this set of images

|  |  |  |  |
| --- | --- | --- | --- |

| Aesthetic Perception: Please rate the beauty of this set of images. |
| --- |
|  |
| Distinctiveness Perception: Please rate the level of coastal uniqueness for this set of images. |
|  |

1. Please rate this set of images

|  |  |  |  |
| --- | --- | --- | --- |

| Aesthetic Perception: Please rate the beauty of this set of images. |
| --- |
|  |
| Distinctiveness Perception: Please rate the level of coastal uniqueness for this set of images. |
|  |

1. Please rate this set of images

|  |  |  |  |
| --- | --- | --- | --- |

| Aesthetic Perception: Please rate the beauty of this set of images. |
| --- |
|  |
| Distinctiveness Perception: Please rate the level of coastal uniqueness for this set of images. |
|  |

1. Please rate this set of images

|  |  |  |  |
| --- | --- | --- | --- |

| Aesthetic Perception: Please rate the beauty of this set of images. |
| --- |
|  |
| Distinctiveness Perception: Please rate the level of coastal uniqueness for this set of images. |
|  |

1. Please rate this set of images

|  |  |  |  |
| --- | --- | --- | --- |

| Aesthetic Perception: Please rate the beauty of this set of images. |
| --- |
|  |
| Distinctiveness Perception: Please rate the level of coastal uniqueness for this set of images. |
|  |

1. Please rate this set of images

|  |  |  |  |
| --- | --- | --- | --- |

| Aesthetic Perception: Please rate the beauty of this set of images. |
| --- |
|  |
| Distinctiveness Perception: Please rate the level of coastal uniqueness for this set of images. |
|  |

1. Please rate this set of images

|  |  |  |  |
| --- | --- | --- | --- |

| Aesthetic Perception: Please rate the beauty of this set of images. |
| --- |
|  |
| Distinctiveness Perception: Please rate the level of coastal uniqueness for this set of images. |
|  |

1. Please rate this set of images

|  |  |  |  |
| --- | --- | --- | --- |

| Aesthetic Perception: Please rate the beauty of this set of images. |
| --- |
|  |
| Distinctiveness Perception: Please rate the level of coastal uniqueness for this set of images. |
|  |

1. Please rate this set of images

|  |  |  |  |
| --- | --- | --- | --- |

| Aesthetic Perception: Please rate the beauty of this set of images. |
| --- |
|  |
| Distinctiveness Perception: Please rate the level of coastal uniqueness for this set of images. |
|  |

1. Please rate this set of images

|  |  |  |  |
| --- | --- | --- | --- |

| Aesthetic Perception: Please rate the beauty of this set of images. |
| --- |
|  |
| Distinctiveness Perception: Please rate the level of coastal uniqueness for this set of images. |
|  |

1. Please rate this set of images

|  |  |  |  |
| --- | --- | --- | --- |

| Aesthetic Perception: Please rate the beauty of this set of images. |
| --- |
|  |
| Distinctiveness Perception: Please rate the level of coastal uniqueness for this set of images. |
|  |

1. Please rate this set of images

|  |  |  |  |
| --- | --- | --- | --- |

| Aesthetic Perception: Please rate the beauty of this set of images. |
| --- |
|  |
| Distinctiveness Perception: Please rate the level of coastal uniqueness for this set of images. |
|  |

1. Please rate this set of images

|  |  |  |  |
| --- | --- | --- | --- |

| Aesthetic Perception: Please rate the beauty of this set of images. |
| --- |
|  |
| Distinctiveness Perception: Please rate the level of coastal uniqueness for this set of images. |
|  |

1. Please rate this set of images

|  |  |  |  |
| --- | --- | --- | --- |

| Aesthetic Perception: Please rate the beauty of this set of images. |
| --- |
|  |
| Distinctiveness Perception: Please rate the level of coastal uniqueness for this set of images. |
|  |

1. Please rate this set of images

|  |  |  |  |
| --- | --- | --- | --- |

| Aesthetic Perception: Please rate the beauty of this set of images. |
| --- |
|  |
| Distinctiveness Perception: Please rate the level of coastal uniqueness for this set of images. |
|  |

1. Please rate this set of images

|  |  |  |  |
| --- | --- | --- | --- |

| Aesthetic Perception: Please rate the beauty of this set of images. |
| --- |
|  |
| Distinctiveness Perception: Please rate the level of coastal uniqueness for this set of images. |
|  |

1. Please rate this set of images

|  |  |  |  |
| --- | --- | --- | --- |

| Aesthetic Perception: Please rate the beauty of this set of images. |
| --- |
|  |
| Distinctiveness Perception: Please rate the level of coastal uniqueness for this set of images. |
|  |
